# Integrated multi-omics reveal important roles of gut contents in intestinal ischemia–reperfusion induced injuries in rats

**DOI:** 10.1038/s42003-022-03887-8

**Published:** 2022-09-09

**Authors:** Die Dai, Fandie Dai, Jingchao Chen, Menglu Jin, Mingyue Li, Desheng Hu, Zhi Liu, Zunjian Zhang, Fengguo Xu, Wei-Hua Chen

**Affiliations:** 1 Key Laboratory of Molecular Biophysics of the Ministry of Education, Hubei Key Laboratory of Bioinformatics and Molecular-Imaging, Center for Artificial Intelligence Biology, Department of Bioinformatics and Systems Biology, College of Life Science and Technology, Huazhong University of Science and Technology, Wuhan, China; 2 Applied Psychology Institution, China University of Geosciences, Wuhan, China; 3 College of Life Science, Henan Normal University, Xinxiang, China; 4 Department of Integrated Traditional Chinese and Western Medicine, Union Hospital, Tongji Medical College, Huazhong University of Science and Technology, Wuhan, China; 5 Department of Biotechnology, Huazhong University of Science and Technology, College of Life Sciences and Technology, Wuhan, China; 6 Key Laboratory of Drug Quality Control and Pharmacovigilance, Ministry of Education, China Pharmaceutical University, Nanjing, China; 7 Jiangsu Key Laboratory of Drug Screening, China Pharmaceutical University, Nanjing, China; 8 State Key Laboratory of Natural Medicine, China Pharmaceutical University, Nanjing, China

**Keywords:** Systems analysis, Gastroenterology

## Abstract

Intestinal ischemia–reperfusion (IIR) is a life-threatening clinical event with damaging signals whose origin and contents are unclear. Here we observe that IIR significantly affect the metabolic profiles of most organs by unbiased organ-wide metabolic analysis of gut contents, blood, and fifteen organs in rats (*n* = 29). Remarkably, correlations between gut content metabolic profiles and those of other organs are the most significant. Gut contents are also the only ones to show dynamic correlations during IIR. Additionally, according to targeted metabolomics analysis, several neurotransmitters are considerably altered in the gut during IIR, and displayed noteworthy correlations with remote organs. Likewise, metagenomics analysis (*n* = 35) confirm the effects of IIR on gut microbiota, and identify key species fundamental to the changes in gut metabolites, particularly neurotransmitters. Our multi-omics results establish key roles of gut contents in IIR induced remote injury and provide clues for future exploration.

## Abstract (web summary)

Die Dai et al. evaluate changes in the metabolomic and gut microbiome in response to experimental intestinal ischemia reperfusion (IIR) injury in rats. Their results provide further insight into how gut contents contribute to widespread injury in IIR patients.

## Introduction

Intestinal ischemia-reperfusion (IIR) is a life-threatening clinical event in many diseases that affect infants, children, and adults^1–4^. It is a result of both surgical and non-surgical approaches such as abdominal aortic aneurysm surgery^5^, cardiopulmonary bypass^5^, small bowel transplantation^6^, acute mesenteric ischemia^7^, neonatal necrotizing enterocolitis^8^, trauma^6^, and septic shock^9^. The primary clinical characteristic of these diseases is the interruption of blood supply, which causes ischemic impairment^10^. Paradoxically, restoring of blood flow does not alleviate the symptoms of the injury; on the contrary, it magnifies the damage drastically. Most astonishingly, IIR substantially impacts the integrity and function of remote organs (the non-ischemic organs), and if severe enough, it can clinically result in systemic inflammatory response syndrome and multiple organ dysfunction syndrome^4^, which account for 30–40% of in-hospital mortality^11^.

In recent years, researchers have advanced several hypotheses on the origin and/or nature of the damaging signals to remote organs during IIR, including intestinal barrier dysfunction^12,13^, inflammatory cytokines release, oxygen-free radical formation^14^, complement activation, microvasculature damage, and neutrophil infiltration^3,15,16^. Several research groups have also investigated the roles of gut microbiota in IIR^17–19^. Per ref. ^17^ found that depleting gut commensal bacteria attenuates IIR-induced injury, while ref. ^18^ established the essential role of commensal bacteria in epithelial restitution after IIR. Collectively, many factors, including Toll-like receptors, oxidative stress and nitric oxide, have been reported to be associated with intestinal bacteria during IIR^19^. However, a few questions remain to be answered: (1) what are the primary source and contents of the damaging signal(s) to remote organs during and after IIR? (2) what route do these signals take to remote organs? (3) what roles do the signals play after they reach remote organs?

To answer these questions, we performed a systemic, time-resolved multi-omic analysis of gut contents, blood, bronchoalveolar lavage fluid (BALF) and fifteen organs during IIR using a rat model. Our results support a gut translocation model in which gut contents leaked into peripheral blood because of a compromised gut barrier during IIR and then to remote organs, causing inflammatory responses. Additionally, we found that several neurotransmitter-related gut metabolites changed significantly during IIR, which could influence remote organs through the enteric nervous system. We also profiled the gut microbiota during IIR, and identified a few key species that underly the changes in metabolites and neurotransmitters. In summary, we provide evidence that gut contents, as the major source of IIR, caused damage to remote organs and established their putative routes. Our results warrant further research on the impact of gut-derived metabolites, neurotransmitters, and microbes.

## Results

### Gut contents leaked into remote organs during IIR because of a compromised intestinal barrier

**Fig. 1:**
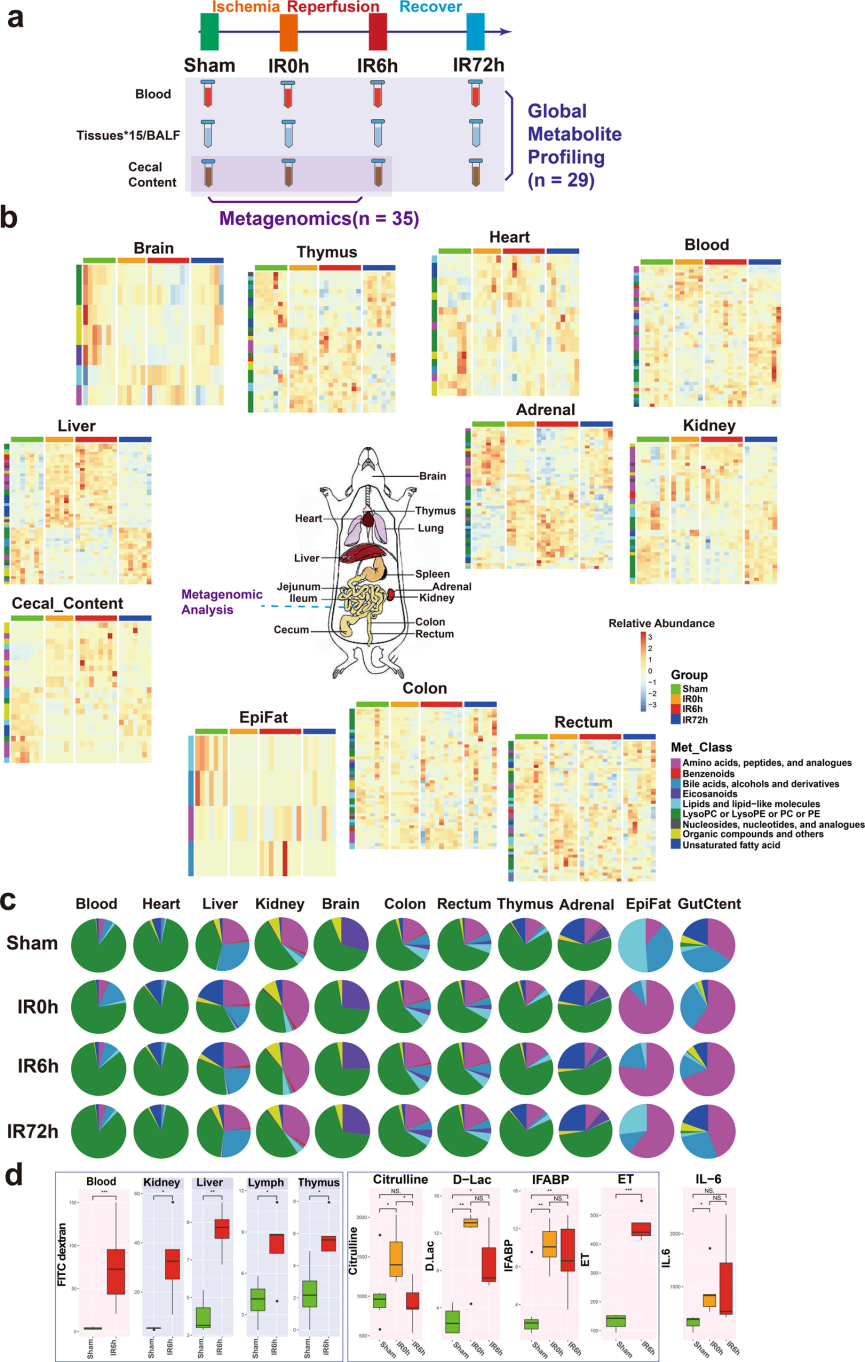
Combination of metagenomic analysis and global metabolite profiling of rat blood/tissues/cecal content reveals common and tissue-specific metabolic signatures during intestinal ischemia and reperfusion (IIR) period. **a** The overall experimental design of this study. Blood, BALF, 15 tissues, and cecal content were collected from rats in Sham, IR0h (the end of 2 h ischemia period), IR6h (reperfusion for 6h) and IR72h (reperfusion for 72h) Groups. Global metabolite profiling was performed for all the samples (*n* = 29). Metagenomic analysis was further implemented for cecal contents (*n* = 35). **b** Metabolic profiles of blood, cecal content, and tissues are significantly influenced by IIR (eleven in all). Each tile represents a metabolite; each column represents a rat. Columns are grouped by time-point during IIR. Row-side colors represent metabolite classes. EpiFat epididymal fat. The units for the heatmap color key represent the relative abundance. **c** Metabolite class compositions in different organs (row) during IIR (column); shown here are the overall relative metabolite masses (sum of standardized abundances) by metabolite classes. GutCtent: cecal content. **d** Some bowel barrier damage-related biomarkers were leaked to blood and remote organs due to intestinal barrier damage, including fluorescein isothiocyanate (FITC)-dextrans, citrulline, d-lactic acid (d-Lac), intestinal-type fatty acid-binding protein (IFABP), and endotoxin (ET); the leakage induced increased level of IL-6 in blood, an inflammatory marker. Background color: red – blood, gray – selected remote organs. The box represents the median, 25th, and 75th percentiles and the error bars indicate the 5th and 95th percentiles. Between-group comparisons were performed using the Wilcoxon test; *p < 0.05; **p < 0.01; ***p < 0.001; NS not significant.

To explore the key roles gut contents play in IIR-induced injury, we carried out a series of multi-layer and multi-angle experiments. Our overall experimental design is shown in Fig. 1a. We first explored unbiased organ-wide metabolic profiling for gut contents, blood, BALF and 15 organs in rats (*n* = 29) to understand the impact of IIR on gut contents and remote organs (Fig. 1b, c). Then we used metagenomics analysis (*n* = 35) to explore the taxonomic and functional characterization alteration of the gut microbiome during IIR.

To confirm previous findings that IIR increases gut permeability by compromising the intestinal barrier^12,13^, we fed Fluorescein isothiocyanate (FITC)-dextran to rats through gavage and quantified its levels in serum and a few tissues during IIR. FITC-dextran is widely used as a marker of mucosal barrier dysfunction and paracellular transport^20^. We also quantified the concentrations of D-lactic acid, fatty acid-binding proteins (IFABPs), citrulline and endotoxins in blood during IIR. D-lactic acid is a fermentation product of various gut bacteria. It has been shown to enter the bloodstream when intestinal permeability increased^21^. IFABPs are small cytosolic water-soluble proteins present in mature enterocytes and reportedly reflect the physiological turnover rate of enterocytes, the increase of which is linked to intestinal epithelial cell damage^22^. Circulating citrulline, an amino acid not incorporated in proteins is mainly produced by enterocytes and is frequently suggested as a biomarker of remnant small bowel mass and function^23^. Endotoxins are constituents of the outer membrane of gram-negative bacteria^24^; systemic endotoxemia is also associated with increased intestinal permeability. These selected substances are representatives of the broad spectrum of gut contents and thus ideal markers for intestinal damage and gut translocation.

As expected, we detected significant changes in the concentrations (*p* values < 0.05, Wilcoxon test) of most of these substances in the blood and a few of them in several tissues (see Fig. 1a for the overall experimental design and Fig. 1d and Supplementary Fig. 1 for the results), suggesting that IIR caused damage to the intestinal barrier, resulting in gut contents leaking into the blood and remote organs. Reduced plasma citrulline levels have only been recognized recently as a biomarker of significantly reduced enterocyte mass and function in different disease states in humans^23^. Here, we detected reduced levels of citrulline in IIR6h compared to the Sham Group, though with no significance. We further confirmed damage to the intestinal barrier (Supplementary Fig. 2) and remote organs (Supplementary Figs. 3–5) using haematoxylin and eosin staining. Levels of the inflammation marker IL-6 increased in the blood (Fig. 1d). We also revealed that several oxidative stress indices, including malondialdehyde, superoxide dismutase and myeloperoxidase, increased in remote organs (Supplementary Fig. 1). Together, our results suggest that gut contents can leak into the blood and remote organs due to compromised intestinal barrier and contribute to IIR-induced damages.

### IIR altered the metabolic profiles of gut contents and remote organs

To provide an unbiased view of body-wide metabolic changes during IIR, we performed global metabolomics profiling using both liquid chromatography-mass spectrometry (LC/TOF-MS) and gas chromatography-mass spectrometry (GC/MS) on blood, gut (cecal) contents, BALF and 15 organs including liver, kidney, adrenal glands, thymus, heart, jejunum, ileum, colon, rectum, brain, epididymal fat (EpiFat), lung, lymph, spleen and subcutaneous fat (SubFat) (Fig. 1a). To check whether the overall metabolic profiles (i.e., features and their relative abundances) of these organs significantly changed during IIR, we applied a multiblock method^25–27^ to integrate metabolomic profiles from both LC/MS and GC/MS. And a PCA2Tree tool^28^ was used to visualize the relationships between the metabolic profiles at four different time points for all organs. PCA2Tree generates a dendrogram from scores in principal component analysis (PCA) using Mahalanobis distances and reports *p* values for the null hypothesis at all internal branches; *p* values less than 0.05 indicated a statistically significant difference.

**Fig. 2:**
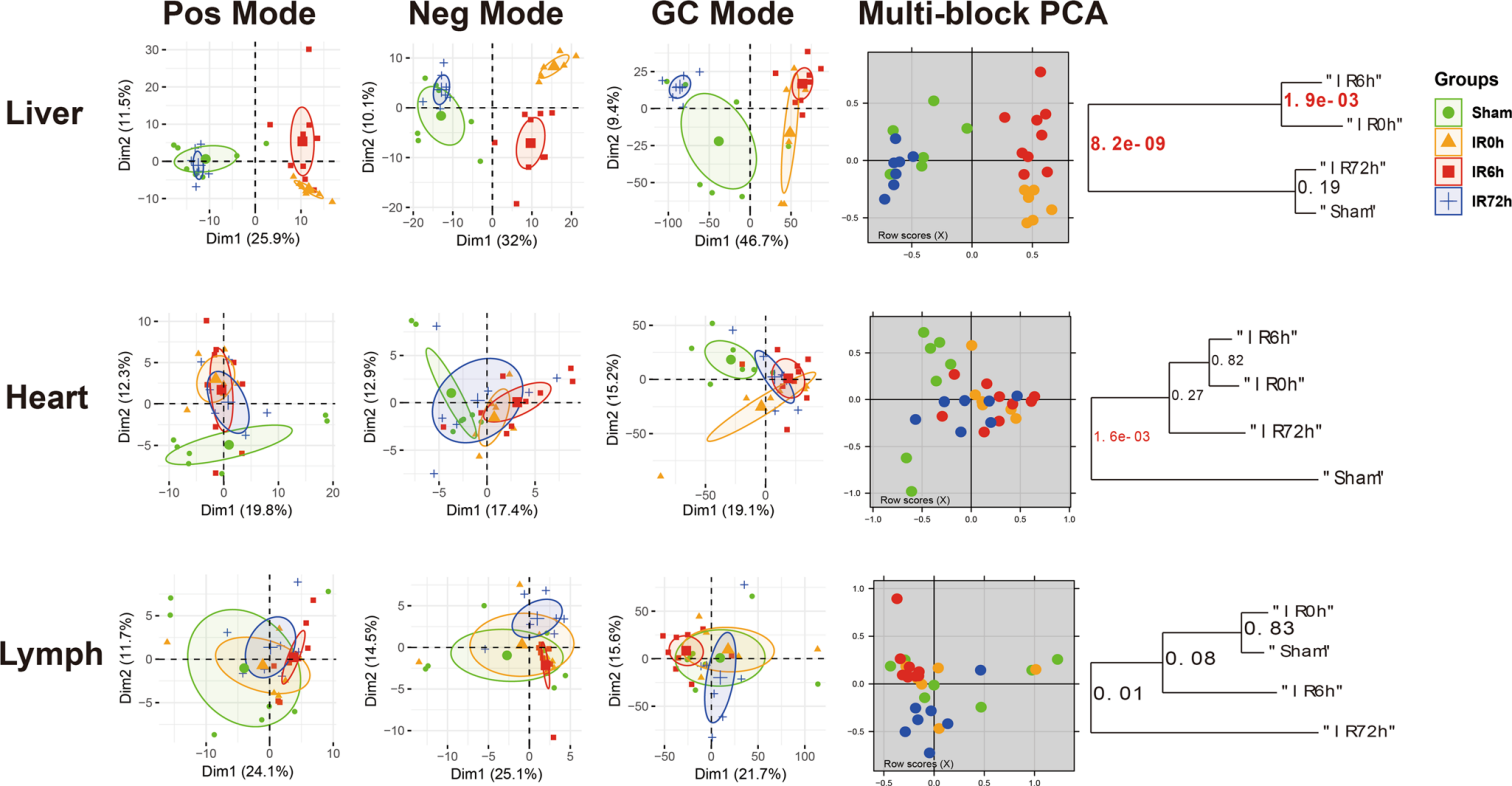
IIR affected metabolomic profiles of intestinal and remote organs that showed three distinct patterns during IIR (selected representatives: liver, heart, lymph). The first three columns are the principal component analysis (PCA) score plots derived from the LC/MS (ESI+), LC/MS (ESI−), and GC/MS metabolite data, respectively. And the fourth column illustrates the multiblock principal component analysis of the data fusion of LC/MS and GC/MS. Plots in the fifth column are metabolic tree diagrams corresponding to the multiblock-PCA score plots using Mahalanobis distances, with *p* values for the null hypothesis shown at each branch. The first two principal components (Dim1 and Dim2) were estimated as sufficiently well represented by the principal plan generated by each PCA. The value of *p* < 0.01 was considered to be significantly different, which were marked in red. Green symbols correspond to Sham groups, yellow symbols to IR0h groups, red ones to IR6h groups and blue ones to IR72h groups. Pos mode: liquid chromatography-mass spectrometry (ESI+); Neg mode: liquid chromatography-mass spectrometry (ESI−); GC mode: gas chromatography-mass spectrometry. Shown here are representatives of three patterns we have identified.

We found that IIR indeed induced metabolic changes in remote organs and identified three distinct patterns (Fig. 2). First, cecal contents and seven organs, including the liver, kidney, rectum, adrenal, thymus, colon, and brain, displayed a stress-recovery pattern. As shown in Supplementary Fig. 6, the metabolic profiles of these samples changed significantly at IR0h (2 h after ischemia) and/or IR6h (6 h after reperfusion) compared to Sham (control group; *P* < 0.01) and were restored to normal at IR72h (72 h after reperfusion). Colon and brain also belonged to this pattern with a different mode (Supplementary Fig. 7). Second, blood and two organs, including the heart and epididymal, showed a stress-non-recovery pattern (Supplementary Fig. 7): their metabolic profiles differed significantly between Sham and IIR72h, indicating a long-lasting effect on these organs.

Third, BALF and six organs, including jejunum, ileum, lung, lymph, spleen, and subcutaneous fat showed no noteworthy changes in metabolic profiles during IIR (Supplementary Fig. 8). These results suggest that our methods could capture metabolic dynamics during IIR. In the subsequent analysis, we focused on the nine organs (liver, kidney, rectum, adrenal glands, thymus, colon, brain, heart, and epididymal), blood and cecal contents that experienced drastic changes in their metabolic profiles (those in the stress-recovery pattern and stress-non-recovery pattern) during IIR and excluded the other six organs (jejunum, ileum, lung, lymph, spleen, and subcutaneous fat) and BALF that showed no notable changes in metabolic profiles during IIR.

We focused on the features (metabolic fragments, Supplementary Data 1) that displayed significant differences in the multiblock analysis (selected using the selectVar function in the partial least squares discriminant analysis (sPLS-DA)^26^) and identified their corresponding metabolites (see Methods). We identified 132 metabolites in total from the abovementioned nine organs, blood, and cecal contents, including 32 amino acids, peptides, and analogs; 5 benzenoids; 7 bile acids, alcohols, and derivatives; 5 Eicosanoids; 18 lipids and lipid-like molecules; 25 lysophosphatidylcholine or lysophosphatidylethanolamine or phosphatidylcholine or phosphatidylethanolamine; 5 nucleosides, nucleotides, and analogs; 25 organic compounds and others and 10 unsaturated fatty acid (Supplementary Fig. 9 and Supplementary Data 1; see Methods for details). The liver presented the most significantly changed metabolites, followed by adrenal glands, with the epididymal fat displaying the least altered metabolites (Supplementary Fig. 10a). The class distributions of the identified differential metabolites were similar across tissues except brain, gut content and epididymal fat (Supplementary Fig. 10b, c). The metabolic contents of an organ, i.e. the metabolite classes and their relative abundances, often showed distinct characteristics from others and changed during IIR (see Fig. 1c and Supplementary Data 2 for the data). Specifically, amino acids and their derivatives increased during ischemia/reperfusion injury in colon, gut content, rectum, liver, thymus, and kidney, but not in the adrenal glands. Owing to the limitations of metabolomics technology and the peculiarity of epididymal fat samples, we identified only four metabolites as significant ones. Therefore, amino acids may not be the dominant metabolites in this tissue. Similarly, unsaturated fatty acids decreased during damage (IIR0h and IIR6h) in gut content and many tissues, like colon, rectum and thymus, whereas increased in adrenal glands, liver and heart. Correlations between metabolites can reflect the synchronization of individual cells in tissues. Metabolites with positive correlations possibly belong to associated pathways, having common functions or origins, whereas negative correlations between metabolites probably reflect incompatible pathways^29^. Astonishingly, the inter-tissue correlation analysis showed extensive negative correlations between unsaturated fatty acids and amino acids (especially tyrosine, phenylalanine, tryptophan, leucine and lysine) in gut content, blood, and most organs (adrenal glands, kidney, thymus, colon, and rectum) (Supplementary Fig. 11).

Pathway enrichment analysis showed that pathways involved in the biosynthesis of amino acids, 2-oxocarboxylic acid metabolism, biosynthesis of unsaturated fatty acids, bile secretion, phenylalanine, tyrosine and tryptophan biosynthesis ranked top (Fisher’s exact test) in most organs (Supplementary Figs. 12, 13). Of these, phenylalanine, tyrosine, and tryptophan are aromatic amino acids that can serve as precursors for neurotransmitters, such as serotonin, dopamine, and norepinephrine^30,31^. Our results suggest that IIR can trigger changes in neurotransmitter metabolism of remote organs. Other inflammation-related pathways, like the mechanistic target of the rapamycin signaling pathway, inflammation mediator regulation of transient receptor potential channels, and eicosanoids changed markedly in several remote organs (Supplementary Figs. 12,13), indicating that inflammatory processes were modulated during IIR. In summary, multiple pathways in intestinal tissues, gut contents, and remote organs were altered during IIR.

### Gut contents play critical roles in IIR-induced damages to remote organs

We next compared metabolic profiles between organs and sought to identify the primary source of IIR-caused damaging signals. We speculated that the metabolic profiles of the source organ would correlate most with other organs, and these correlations would show dynamic changes during IIR.

**Fig. 3:**
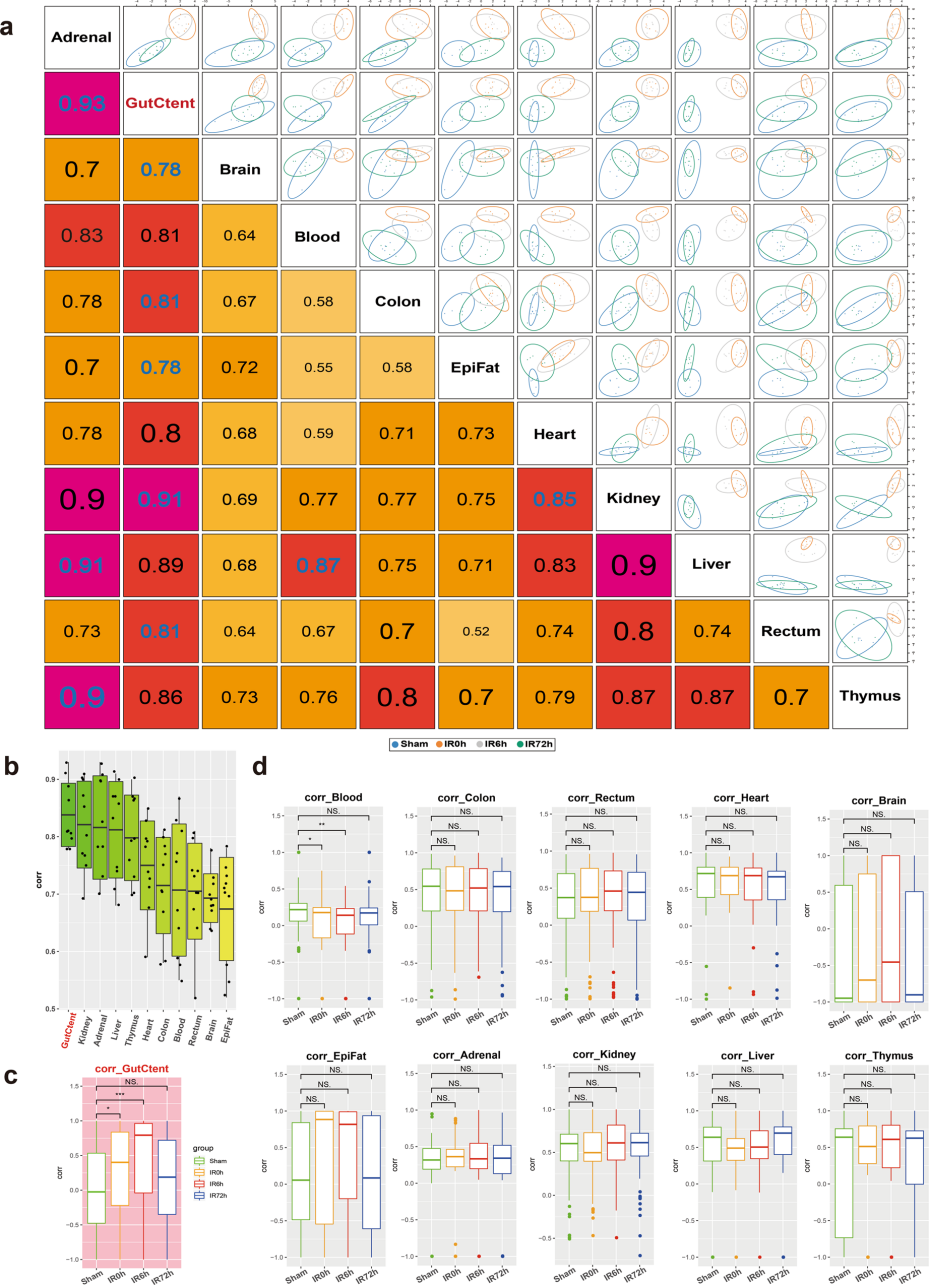
Cross-tissue metabolite correlations revealed the vital role of gut content in the impact of remote organs caused by IIR. **a** Correlations between tissues based on identifying a multi-tissue signature composed of highly correlated features across different tissues. The larger the correlation coefficient, the stronger the relevancy between the tissues. **b** Boxplots summarizing the correlations in **a** show that gut contents have the highest overall correlation with others. **c**, **d** Pairwise Spearman correlations as a function of time during IIR. Gut contents (**c**) were the only organ that showed dynamic correlations with other tissues. Between-group comparisons were performed using the Wilcoxon test, *n* = 7 biologically independent animals in Sham group and *n* = 6 in IIR0h group, *n* = 9 in IIR6h group, *n* = 7 in IIR72h group; *p < 0.05; **p < 0.01; ***p < 0.001; NS not significant. The box represents the median, 25th, and 75th percentiles and the error bars indicate the 5th and 95th percentiles.

We first calculated pairwise correlations of the metabolic profiles across organs (Fig. 3a) using the R package “mixOmics”^26^. Accordingly, gut contents displayed the most significant overall correlation co-efficiencies with other organs (Fig. 3b), followed by kidney, adrenal glands, liver, and thymus. We then calculated the correlations as a function of time during the IIR experiments and found that gut contents were the only one that showed dynamic changes in its correlation co-efficiencies with other tissues (Fig. 3c, d). These correlations increased considerably at IR0h compared to Sham and rose further at IR6h, consistent with our observation that gut contents leaked into remote organs during IIR and decreased to comparable levels to that of the Sham group at the end of the recovery stage (IR72h, Fig. 3c). Similar patterns were not observed in other tissues (Fig. 3d). Furthermore, we identified more correlations between gut contents and remote organs than between intestinal tissues (colon and rectum) and remote organs (Supplementary Fig. 14). Together, our results indicate that gut contents are crucial to IIR induced damages to remote organs.

We then examined the metabolites underlying the correlations between gut contents and remote organs. As shown in Supplementary Fig. 15, nine gut content metabolites, including leucine, cyclic phosphatidic acid (CPA (18:1(9Z)/0:0)), phenylalanine, acetylcholine, lysine, linoleic acid, alpha-linolenic acid, leucyThreonine, and hypotaurine correlated substantially with remote organs. Surprisingly, most of them are either neurotransmitters or associated with their biosynthesis or regulations. For example, acetylcholine is a neurotransmitter^32^. Phenylalanine is an aromatic amino acid and a precursor for neurotransmitters, such as serotonin, dopamine, and others^30^. Lysine is a key precursor for the de novo synthesis of glutamate, which is the most important excitatory neurotransmitter in the mammalian central nervous system^33^. Linoleic acid is a long-chain polyunsaturated fatty acid, which has been verified as capable of inducing neurotransmitter release^34^. These results suggest that gut-derived neurotransmitters also contribute to IIR-induced damages to remote organs.

### Alteration in gut content metabolome during IIR points to an impairment in intestinal neuroendocrine function

Having established that gut contents contribute notably to IIR-induced damages, we further explored the impact of their alteration during IIR and their putative roles in IIR-induced injury to remote organs.

**Fig. 4:**
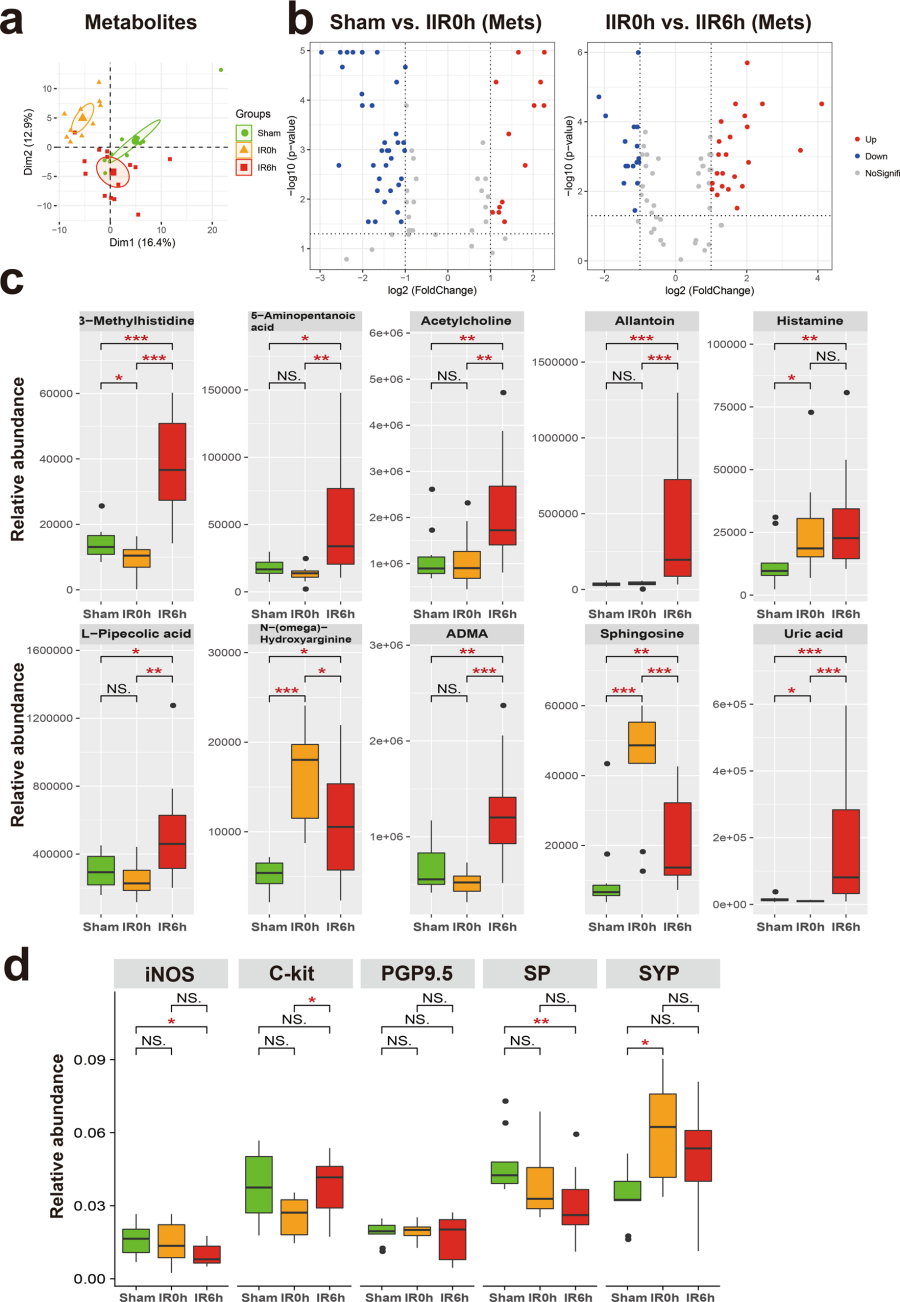
Significantly altered metabolites and markers for neuroendocrine system in the gut during IIR. **a** Principal component analysis (PCA) score plot derived from metabolites abundance. **b** The volcano plots displayed the statistical significance (*P* value) versus the magnitude of change (Log2 fold change). Features with Wilcoxon test *p* value <0.05 and absolute Log2 fold change (Log2FC) >1 were deemed to be significantly different. **c** Shown here are gut metabolites that were significantly altered during IIR and were not returned to normal levels at IIR6h, i.e., significantly different between IIR6h compared to Sham. Between-group comparisons were performed using the Wilcox test, *n* = 10 biologically independent animals in Sham group and *n* = 15 in IIR0h group, *n* = 10 in IIR6h group; *p < 0.05; **p < 0.01; ***p < 0.001; NS not significant. **d** Immunohistochemical level of several molecular markers for the intestinal neuroendocrine system significantly changed during IIR: nitric oxide synthase (iNOS), C-Kit, synaptophysin (SYP), protein gene product 9.5 (PGP9.5), and substance P (SP). Between-group comparisons were performed using the Wilcoxon test, *n* = 3 biologically independent animals; *p < 0.05; **p < 0.01; NS not significant. The box re*p*resents the median, 25th, and 75th percentiles and the error bars indicate the 5th and 95th percentiles.

We first identified metabolites that changed notably during IIR (Fig. 4a, b and Supplementary Data 3–6), and classified them into two groups according to whether their abundances differed considerably between IR6h and Sham (referred to as the Unrecovered group) or not (referred to as the Recovered group). We further divided the Recovered metabolites into two subgroups based on whether they were up- or downregulated at IR0h, and referred to them as Recovered_UpDown and Recovered_DownUp, respectively. The first subgroup (Recovered_UpDown) included l-arginine, 4-guanidinobutyric acid, d-fructose, cellobiose, d-maltose, d-lactose, alpha-d-glucose, stachyose, maltotriose, 3-phenylpropanoic acid, and kynurenic acid (Supplementary Fig. 16), while the second (Recovered_DownUp) consisted of flavin adenine dinucleotide, uridine monophosphate, guanosine monophosphate, l-aspartate, and arachidonic acid (Supplementary Fig. 17). Most of these molecules participate in carbohydrate metabolism, the probable sources of carbon and energy for intestinal bacteria. L-arginine is also a possible carbon and nitrogen source. Their significant changes at IR0h and subsequent recovery at IR6h are likely caused by the fluctuations in the metabolic capabilities of gut microbiota in response to oxygen supplies during IIR. For example, large quantities of carbon, nitrogen, and energy accumulated during ischemia, probably due to metabolism arrest, and then were used by intestinal bacteria in the following reperfusion period.

We also divided the UnRecovered metabolites into two subgroups based on whether they were up- or down-regulated at IR6h and labeled these groups as Unrecovered_Up and Unrecovered_Down, respectively. The UnRecovered_Down subgroup included nicotinamide adenine dinucleotide, uridine diphosphate glucose (UDP-glucose), UDP, UDP-N-acetylglucosamine, cytidine diphosphate, citrate, and isocitrate (Supplementary Fig. 18). UDP-glucose^35^, as an activated form of glucose, is a nucleotide sugar involved in glycosyltransferase reactions in metabolism. It is a precursor of glycogen, sucrose, lipopolysaccharides and glycosphingolipids. The abnormal variations in the quantities of these molecules could reflect disturbances in carbohydrate metabolism.

The Unrecovered_Up subgroup comprised uric acid, allantoin, L-pipecolic acid, 5-aminopentanoic acid, 3-methylhistidine, histamine, acetylcholine, sphingosine, *N*^G^,*N*^G^-dimethyl-L-arginine (ADMA), and *N*-(omega)-Hydroxyarginine (Fig. 4c). Outstandingly, several of them are associated with neurotransmitters. For example, acetylcholine and histamine are neurotransmitters. ADMA is an endogenous nitric oxide synthase inhibitor,^36^ and *N*-(omega)-Hydroxyarginine is an intermediate in the biosynthesis of nitric oxide from l-arginine^37^. They are both linked to nitric oxide, which is a signaling molecule in the nervous system^38^. 5-Aminopentanoic acid is a methylene homolog of gamma-aminobutyric acid, an inhibitory neurotransmitter in the human cerebral cortex^39^, and sphingosine is a sphingolipid metabolite that can exert vital neuron-specific functions, such as the regulation of neurotransmitter release^40^.

To further explore the influence of IIR on neurotransmitter levels and intestinal neuroendocrine function, we first performed a targeted metabolomics analysis using LC–MS/MS. Several neurotransmitters and related metabolites, including γ-aminobutyric acid, serotonin, epinephrine, and glutamine changed (Supplementary Fig. 19). Next, we utilized an immunohistochemical approach to assess the influence of IIR on the intestinal neuroendocrine system. We applied several antibodies, including nitric oxide synthase, C-Kit, synaptophysin, protein gene product 9.5, and substance P. They are commonly used molecular markers of the neuroendocrine system^41^ and enteric nervous system^42–45^. As expected, these markers were drastically altered during IIR (Fig. 4d and Supplementary Fig. 20), indicating an impairment in intestinal neuroendocrine function.

Together, our results suggest that IIR induces complex changes in the gut metabolome. Many of the Recovered metabolites participated in carbohydrate metabolism and energy production, consistent with the stress-recover design of our experiments. However, the functions of the Uncovered metabolites, most of them being neurotransmitters, were more diverse. The latter metabolite types can interact with and subsequently induce changes to the enteric nervous system and then influence remote organs. The long-lasting changes in their abundance after reperfusion indicate that they possibly contribute to the long-term impact of IIR on remote organs. Compared to their Recovered counterparts, UnRecovered metabolites perhaps also play critical roles in physiological or pathological processes of IIR-induced impairment of remote organs and warrant further investigation.

### Taxonomic and functional alterations in the gut microbiome during IIR

To further explore the alteration of intestinal flora and their associations with gut metabolome, we submitted cecal samples to metagenomic next-generation sequencing, followed by taxonomic analysis using MetaPhlAn2^46^ and functional characterization using HUMAnN2^47^ (see Methods for details).

**Fig. 5:**
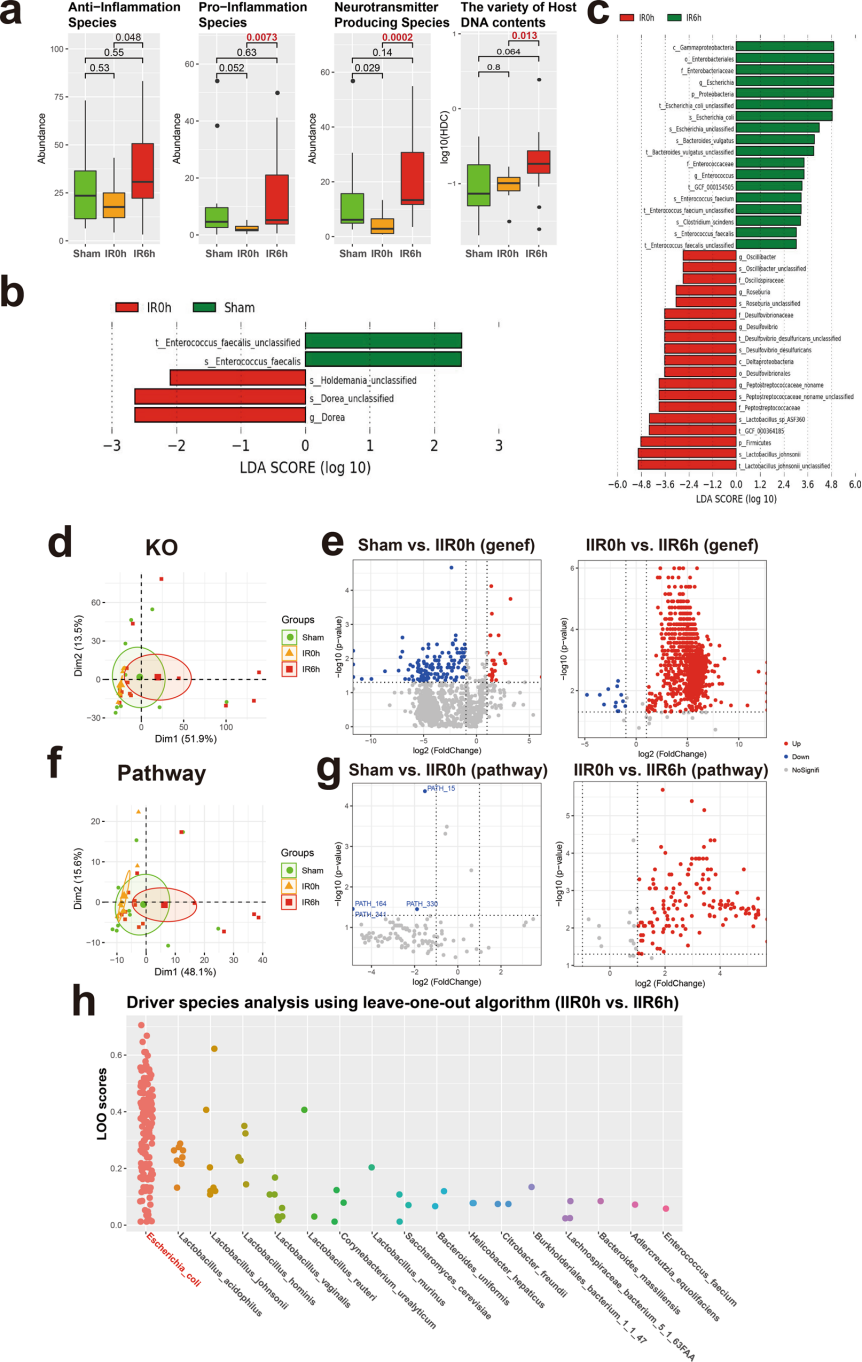
Taxonomic and functional analysis of gut microbiome during IIR. **a** Overall abundance changes for species with known functions and/or characteristics, including anti-inflammation, pro-inflammation, and neurotransmitter-producing capacity; the last panel shows the proportion of host DNA contents (HDC) that serves as a gut metagenomic marker for intestinal barrier integrity (See Methods for details). Between-group comparisons were performed using the Wilcox test, *n* = 10 biologically independent animals in the Sham group and *n* = 15 in the IIR0h group, *n* = 10 in the IIR6h group. The box represents the median, 25th, and 75th percentiles and the error bars indicate the 5th and 95th percentiles. **b**, **c** Differential species between time points as using LEfSe analysis. LDA score threshold of >2.0 and a 0.05 alpha value for the factorial Kruskal–Wallis test was applied to select different bacteria. **d** Functional analysis using KO. PCA analysis on KO profiles; each dot represents a sample (rat). **e** Volcano plots showing differential KO categories between IR0h as compared to Sham and IR6h as compared to IR0h. X-axis: *P* value (Wilcoxon rank-sum test), Y-axis: −Log2 fold change. Each dot represents a KO category; those with *P* values <0.05 and absolute Log2 fold change (Log2FC) >1 were highlighted and deemed to be significantly different. **f**, **g** Functional analysis using microbial pathways; similar to **d**, **e**. **h** Driver species analysis for the metabolic pathways using leave-one-out (LOO) algorithm. Each dot represents a pathway. X-axis: driver species, Y-axis: LOO score; the higher the score, the more important the species is for the pathway. *Escherichia coli* was identified as the strongest driver specie for most of the functional pathways during IIR.

We identified a total of 111 species in all samples (see Fig. 1a for the overall experimental design). We first focused on species with known functions/characteristics, whose information we obtained from large numbers of literature retrieval (listed in Supplementary Data 7). We found that obligate anaerobes increased at IR0h and decreased at IR6h (Supplementary Fig. 21a), consistent with our experimental design showing that oxygen supply decreased during ischemia but leveled up during reperfusion. There showed no significant difference in microbial alpha diversity (Shannon index and Simpson index) between groups (Supplementary Fig. 21b). And beta diversity showed a tendency of separation among microbial communities characterized by PCoA on weighted UniFrac distance (Supplementary Fig. 21b). Remarkably, the overall abundances in pro-inflammation species increased significantly at IR6h (Fig. 5a and Supplementary Data 7), some of which are known to modulate gut inflammation, suggesting that changes in gut microbiota during IIR can, in turn, influence the physiology and function of the gut. We also revealed that neurotransmitter-producing bacteria were considerably affected during IIR; more importantly, their overall abundances were notably higher at IR6h than IR0h, with an increasing trend, though with an insignificant difference, also apparent at IR6h compared to Sham, although the difference was not significant (Fig. 5a). Worth noting is that we only assigned functional characteristics to part of the total 111 species identified in this study. Our results could, therefore, be biased because of such a limitation. Additionally, we found significant differences in HDCs (host DNA contents, an indicator of a compromised intestinal barrier, Fig. 5a) between groups, indicating that intestinal barriers were compromised during IIR^48^.

We used a linear discriminant analysis (LDA) effect size (LEfSe) analysis to identify differential taxa between time points. Five differential taxa were identified at IR0h compared to Sham (Fig. 5b and Supplementary Fig. 21c; LDA score >2.0, *p* < 0.05 with Kruskal–Wallis test, Supplementary Data 8). And 37 differential taxa were identified at IR6h compared to IR0h (Supplementary Data 9). Of these, several beneficial species, including *Lactobacillus johnsonii* and *Lactobacillus sp ASF360*, reduced significantly, with others, including *Escherichia coli* and *Bacteroides vulgatus*, and species in the Enterobacteriaceae and Enterococcaceae families increasing markedly (Fig. 5c and Supplementary Figs. 21c, 22).

As expected, the functional analysis of the sequencing data identified significantly more downregulated genes and pathways than upregulated ones at IR0h compared to Sham (Fig. 5e, g and Supplementary Data 10–15; see Methods for details), suggesting that cutting off blood supply to the gut suppressed the overall activities of gut microbes. Conversely, we identified significantly more upregulated genes and pathways than downregulated ones after blood supply restoration (IR6h compared to IR0h, Fig. 5e, g). Furthermore, PCA analysis established differences in both microbial beta diversities (Supplementary Fig. 21b) and their functional potentials (i.e., genes and microbial pathways, Fig. 5d, f) between IR6h and Sham. Therefore, gut microbes changed significantly during IIR, with some changes not restored after reperfusion. Additionally, functional characterization analysis of gut microbiome revealed that bacterial metabolisms associated with the biosynthesis of neurotransmitters-related metabolites, such as pathway phosphoribosyl pyrophosphate (PRPP)-PWY; histidine and tryptophan biosynthesis pathways; CITRULBIO-PWY: l-citrulline biosynthesis, were significantly augmented (Supplementary Data 13–15). PRPP is a precursor for histidine and tryptophan biosynthesis pathways, which are associated with the neurotransmitter histamine. CITRULBIO-PWY is linked to *N*-(omega)-Hydroxyarginine, which is related to nitric oxide, a signaling molecule in the nervous system. Remarkably, metabolomics analysis identified these neurotransmitter-related molecules to be significantly increased. Hence, we inferred that alterations in gut content metabolites could be the result of abnormal gut microbiota metabolism.

We also uncovered the principal microbial driver species for all the functional pathways using a leave-one-out algorithm^49^ (Supplementary Data 16, 17). Surprisingly, *E. coli*, which increased sharply at IIR6h, was identified as the strongest driver specie for most of the functional pathways (Fig. 5h and Supplementary Fig. 21d). These results suggested a causal relation between *E. coli* and those significantly altered metabolites at IIR6h, which is further supported by several literatures, linking *Enterobacteriaceae*, and more specifically *E. coli*, to IIR associated diseases such as acute surgical abdomen^50^, small bowel transplantation^51^, necrotizing enterocolitis^52–54^, and sepsis^55^. Because IIR-induced remote organ injury is a complex physiological process associated with many factors, systematic and comprehensive explorations are still needed to further validate whether *E.coli* is truly the driver of downstream IIR-instigated complications.

### Inter-omics analysis links gut microbiota to metabolites during IIR

**Fig. 6:**
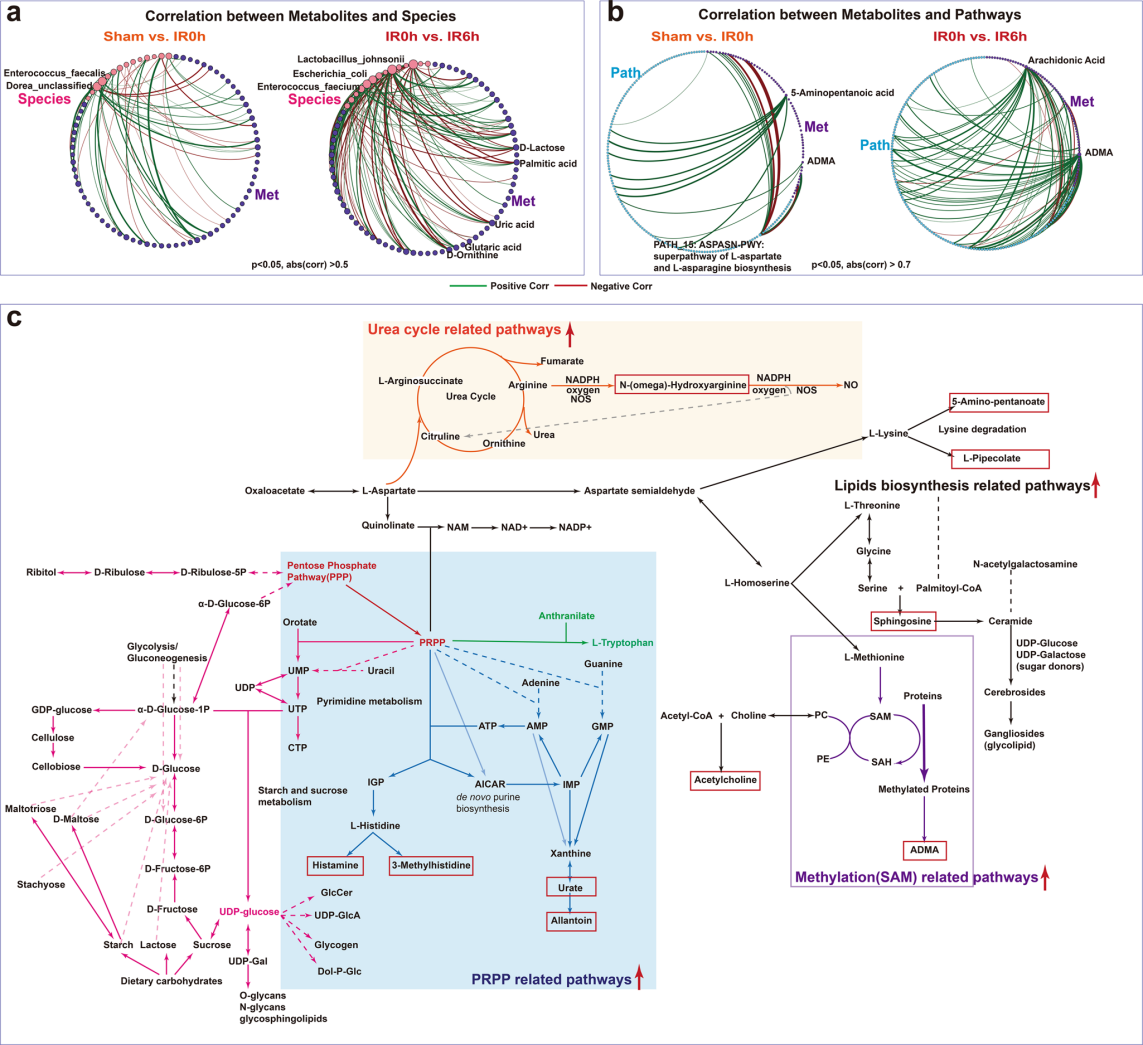
Integrated microbiome-metabolome analysis identified key metabolic pathways and underlying key species. **a**, **b** Significantly more connections (Spearman correlations) were identified between microbial metabolites and species abundance as well as gut pathways during the reperfusion period than that in the ischemia period. **c** A global map linking significantly altered metabolites and pathways and their putative underlying key species. Metabolites in the red boxes were those sharply increased at IR6h compared to Sham. Red arrows indicated that the associated pathways were significantly increased at IIR6h compared to Sham and IIR0h.

To link the altered gut metabolites during IIR to gut microbes, we performed an inter-omics Spearman correlation analysis (see Methods for details). The overall correlation pattern between microbial metabolites and gut pathways during reperfusion was distinctive from that during ischemia (Fig. 6b and Supplementary Fig. 23). We identified more connections between pathways and metabolites (Supplementary Fig. 23), particularly markedly elevated neurologic function-related molecules, like ADMA, 5-aminopentanoic acid, histamine, and acetylcholine during reperfusion. Likewise, we observed more links between gut metabolites and *E. coli*, *L. johnsonii* and several other species in genus *Enterococcus* during reperfusion (Fig. 6a and Supplementary Fig. 24). Furthermore, *E. coli* and species in the genus *Lactobacillus* correlated strongly with gut-derived metabolites in Model-based Integration of Metabolite Observations and Species Abundances 2 (MIMOSA2)^56^ analysis (Supplementary Fig. 25). These results are consistent with the microbial driver analysis that a few key species are probably fundamental to the changes in gut metabolites.

To further explore the influence of intestinal microflora on metabolites at the biotransformation level, we created a global metabolic map connecting significantly altered metabolites at IIR6h (primarily including metabolites in carbohydrate metabolism and several neurotransmitters-related ones, Fig. 6c, Supplementary Fig. 26, and Supplementary Data 13–15) based on known pathways they involved in. As Fig. 6c demonstrates, pathway phosphoribosyl pyrophosphate (PRPP)-PWY (superpathway of histidine, purine, and pyrimidine biosynthesis) played a vital role in linking carbohydrate metabolism with multiple pathways like pyrimidine, purine, and pyridine nucleotide synthesis pathway, histidine and tryptophan biosynthesis pathways. These PRPP-related pathways increased significantly at IIR6h (Supplementary Data 15), and metabolites synthesized from them, including histamine, 3-methylhistidine, uric acid, and allantoin, also rose significantly at IIR6h compared to Sham (Fig. 4c). Moreover, ADMA, N-(omega)-hydroxyarginine and sphingosine underwent significant amplification at IIR6h compared to Sham (Fig. 4c). ADMA is synthesized when arginine residues in proteins are methylated. Methylation-associated pathways increased at IIR6h in this research. N-(omega)-Hydroxyarginine is an intermediate in the biosynthesis of nitric oxide from l-arginine. The corresponding urea cycle-related pathways like CITRULBIO-PWY: l-citrulline biosynthesis were also increased at IIR6h. Sphingosine belongs to lipids, and pathways related to lipids synthesis, like PWY-5138: unsaturated, even-numbered fatty acid and beta-oxidation, widely increased at IIR6h.

In summary, we identified some key species fundamental to the changes in gut metabolites and their putative roles in regulating the metabolism of neurotransmitters. Gut metabolite alterations during reperfusion could be the result of gut bacteria metabolism activation after blood supply restoration.

## Discussion

Gut microbes have been implicated in IIR-induced damages to remote organs. However, it remains unclear if gut contents contribute substantially to these damages and what the molecular nature of the damaging signals and their impacts on remote organs are. In this study, we first confirmed with selected biomarkers that IIR increased gut barrier permeability, caused gut contents to leak into the blood and remote organs, and induced inflammatory responses in the blood. These results support a gut translocation model for IIR-induced damages. Remarkably, we also found injuries in several remote organs by haematoxylin and eosin staining, alongside numerous elevated oxidative stress indices during IIR (Fig. 1d and Supplementary Figs. 1–5).

We performed a time-resolved body-wide metabolic profiling of blood, gut contents, BALF, and fifteen organs during IIR to determine the importance of gut contents in IIR-induced damages. IIR significantly altered the metabolic profiles of most organs. According to cross-correlation analysis, gut content metabolic profiles displayed the most notable overall correlation co-efficiencies with those of other organs (Fig. 3b), and were the only one whose correlations with other tissues changed dynamically (Fig. 3c, d). As a result, we argue that gut content was the primary source of damaging signals to remote organs during IIR.

To further explore how cecal metabolites and microbes altered metabolomes in remote organs, we performed an integrated multi-omics analysis of gut content during IIR in multi-layer and multi-angle. Through metabolomics analysis and evaluation of every identified metabolite in gut contents during IIR, we concentrated on metabolites markedly altered at IIR6h (the injury point) compared to the Sham and IIR0h groups. Outstandingly, most of these metabolites are neurotransmitters related (Fig. 4c), such as acetylcholine, histamine and ADMA. These molecules may contribute to the long-term impact of IIR and paly critical roles in physiological or pathological processes of IIR-induced impairment of remote organs, by e.g., interacting with enteric neurons, as suggested previously^57^. Furthermore, we examined the metabolites fundamental to the correlations between gut contents and remote organs. Astonishingly, most of them, including phenylalanine, linoleic acid and hypotaurine, are either neurotransmitters or associated with their biosynthesis or regulations. Additional analyses using targeted metabolomics and immunohistochemistry validated our finding of changes in neurotransmitters-associated metabolites and molecular markers for the neuroendocrine system, indicating impairment in intestinal neuroendocrine function during IIR. Inter-omics analysis linked differential metabolites in the gut to significantly changed pathways and/or gene functions of gut microbes. While each method has its strengths and limitations, it is fascinating that various statistical methods established similar findings. Gut microbiota has been reported^58^ to closely interact with the body’s neuroendocrine system through the hypothalamic–pituitary–adrenal axis, which is also interrelated with the immune system, gut hormones as well as autonomic nervous systems.

In summary, we revealed that gut contents are critical to IIR-caused remote organ injuries. We performed an integrated multi-omics analysis of gut content during IIR in multi-layer and multi-angle, trying to explore how cecal metabolites and microbes could alter the metabolomes in remote organs. Here we established the routes of gut contents to remote organs, including translocation through compromised gut barrier and influencing the enteric nervous system by neurotransmitters. As IIR-induced remote organs injury is a complex physiological process associated with many factors, systematical and comprehensive explorations are still needed. Our results provide clues for future experiments aiming to illustrate the molecular mechanisms causing the damages. Given that experimental rats are small in size, future efforts are still needed to further explore and validate the study findings.

## Methods

### Animal experiments and sample collection

All animal experiments were approved by the Animal Ethics Committee of Huazhong University of science and technology and were performed in accordance with their guidelines. Male Sprague–Dawley rats (220–260 g body weight) in specific pathogen-free status were purchased from Vital River Laboratory Animal Technology Co., Ltd., Beijing, China. Rats were randomly assigned to the Sham or IIR group, maintained under controlled conditions of temperature(21 ± 1 °C) and relative humidity (&55%) on a 12 h light/12 h dark cycle. Animals were fed ad libitum with food and water for 1 week before experiments and were fasted overnight before the day of the experiment.

For the IIR group, rats were treated with anesthesia with 20% urethane. A midline laparotomy was performed to expose the superior mesenteric artery and occlusion of the superior mesenteric artery was applied with a noncrushing microvascular clamp. After the 120-minute period of ischemia, the micro clamp was removed, and the blood supply was restored. Sham animals were also subjected to laparotomy, but with no occlusion of the superior mesenteric artery. Samples, including Blood, tissues and cecal contents, were immediately collected for the Sham and IIR group at the ending of ischemia (IR0h) and in the period of reperfusion (IR6h, IR72h). On collection, samples were subsequently stored at −80 °C for further analysis.

In total, 64 rats were used in our experiments and a two-stage approach was performed. First, 29 rats (*n* = 7 in Sham, *n* = 6 in IIR0h, *n* = 9 in IIR6h, *n* = 7 in IIR72h) were sacrificed for global metabolite profiling, and then 35 rats (*n* = 10 in Sham, *n* = 15 in IIR0h, *n* = 10 in IIR6h) were used for metagenomic analysis and further validation.

### Biochemical indexes determination and Histological analyses

The biochemical indexes (Citrulline, d-lactic acid, IFABP, IL-6, endotoxin, myeloperoxidase, superoxide dismutase, malondialdehyde) were determined using a BioTek Epoch microplate spectrophotometer (Vermont, USA) and Chemray 240 automated biochemical analyzer (Shenzhen Redu Lifeience and Technology, Shenzhen, China). All assay plates were run according to the manufacturer’s protocol.

### FITC-dextran assay

Four hours prior to operation, rats received a 20 ml/kg dose of 4 kDa fluorescein isothiocyanate (FITC)‐dextran (Sigma‐Aldrich, NSW, Australia, Catalog # 4013) by oral gavage. Blood was collected at IIR 6 h by retro-orbital bleeding and stored in a dark and dry place to protect it from light. Samples were centrifuged at 3000×*g* for 5 min and serum isolated. Then dilute serum samples 1:2 with 1 X PBS. FITC-Dextran was quantified using Spark 10 M microplate spectrophotometer (Tecan, Austria) relative to a standard curve (range 12.5–2500 ug/ml).

### Hematoxylin and eosin staining

The collected tissues were first fixed and then dehydrated and embedded in melted paraffin wax. The obtained block was then mounted on a microtome and cut into thin slices. The slices were affixed to microscope slides with the wax removed. The tissue slices attached to the slides were rehydrated and then stained. The hematoxylin and eosin staining was performed by the first application of haematoxylin mixed with a metallic salt, then removing excess staining with a rinse in a weak acid solution, followed by building in mildly alkaline water, then counterstaining with eosin. Following the eosin stain, the slide was passed through several changes of alcohol to remove all traces of water, then rinsed in several baths of xylene, which clears the tissue and renders it completely transparent. A thin layer of polystyrene mountant was used, followed by a glass cover slip. Then microscopic image acquisition and analysis were performed.

### Immunohistochemical analysis

The paraffin sections of the intestinal were immunostained with antibodies nitric oxide synthase Rabbit pAb (# A0312), C-Kit (#A0357), synaptophysin (#A6344), protein gene product 9.5 (#A19101), substance P (#A13550) (ABclonal Biotech Co., Ltd, Wuhan, China), which were commonly used molecular markers for neuroendocrine system^41^ and enteric nervous system^42–45^. All procedures were performed according to the recommended protocols of Abclonal’s film method.

The immunostaining level was assessed and analysed using the Image-pro Plus 6.0 (IPP 6.0) software^59^. The irregular automated optical inspection function in the software was applied to score and rule out non-target staining. The measurement parameter was integrated optical density. All images obtained from IPP 6.0 were further verified by pathologists. Measurements for three cores per sample were averaged and the mean value of the integrated optical density was considered the final value.

### Metabolome analysis

Coupling chromatography to mass spectrometry (MS) offers a combination of sensitivity and selectivity for complex mixture analyses and has evolved as a critical and indispensable platform in metabolomics. As each detecting method has its biases, a combination of gas chromatography (GC)-MS and liquid chromatography (LC)-MS data provides a more comprehensive and unbiased metabolite profiling.

Serum/tissue extracts/cecal contents were first thawed at 4 °C and then mixed with acetonitrile to perform protein precipitation. The supernatant was obtained after two centrifugations (16,000 rpm, 10 min, 4 °C) and was further divided into two aliquots. One was used for GCMS analysis after the complex derivatization process, i.e., alkylation and silylation, whereas the other was employed directly for UFLC-IT-TOF/MS (ion trap/time-of-flight hybrid mass spectrometry) analysis^60,61^.

For LC/MS analysis, the Phenomenex Kinetex C18 column was used to perform chromatographic separations. Gradient elution was applied with 0.1% formic acid and acetonitrile at a constant flow rate of 0.4 mL/min. The column temperature was kept at 40 °C and the injection volume was 5 μL. Electrospray ionization (ESI) source in both the positive and negative ion mode was used for mass analysis. The detailed MS instrumental parameters were set as previously described^60^. Mass spectrometry operation parameters were set as follows: positive and negative ion modes were both used. And interface voltage was set at 4.5 and −3.5 kV for each mode, respectively. The mass range was scanned from 100 to 1000 m/z using an accumulation time of 20 ms per spectrum. Nitrogen was used as the nebulizer and drying gas, set at a constant flow rate of 1.5 and 10 L/min, respectively. Metabolites were identified by comparing the formulae, mass-to-charge ratio (m/z), and the MS/MS fragmentation information with our labs’ self-built database and the online database, such as LIPID MAPS (http://www.lipidmaps.org), Human Metabolome Database (HMDB; http://www.hmdb.ca), and Metlin database (http://metlin.scripps.edu). For further validation, gut content metabolome were reanalyzed using the Agilent 1290 Infinity LC system (Agilent Technologies, Santa‐Clara, CA) coupled to the AB Triple TOF 5600/6600 system (AB SCIEX, Framingham, MA) with an Acquity UPLC BEH Amide column (2.1 mm × 100 mm, 1.7 μm, Waters).

For GC/MS analysis, helium was used as the carrier gas and the flow rate was 1 mL/min. The oven temperature was programmed at 70 °C for 2 min and then increased at a rate of 10 °C/min to 320 °C, and held at 320 °C for 2 min. And the total run time was 29 min. The mass spectrometer was operated in electron impact mode (70 eV) at full scan mode from m/z 45 to 600 with a scan time of 0.2 s. The injector split ratio was set to 1:50. Metabolites in GC/MS were identified by comparison of mass spectra and retention time with those in the commercial compound libraries, National Institute of Standards and Technology (NIST). And they were further confirmed by comparison with the reference standards in our lab.

### Data preprocessing of metabolomics

Peak deconvolution and alignment for the LC/MS and GC/MS chromatograms were performed using Profiling Solution version 1.1 (Shimadzu, Kyoto, Japan) installed in the instrument. As a result, a matrix containing matched peaks with m/z value, retention time and corresponding intensities was generated and was then exported to an Excel table.

Possible sources of instrumental or reagent contamination were excluded by randomly injecting solvent blanks and checking against the blank runs. The so-called 80% rule was used to retain metabolites detectable in at least 80% of subjects. And 30% rule was applied to remove features with higher relative standard deviation (RSD) in quality control (QC) samples. Furthermore, the missing values were filled with half of the minimum value in the dataset. Additionally, the total area normalization was performed and the relative abundances were obtained.

### Quantification of neurotransmitters in gut content

LC–MS/MS analysis was performed on an Agilent 1290 Infinity UHPLC coupled to an AB Sciex Qtrap ® 5500 MS/MS using an Acquity UPLC™ BEH Amide column (2.1 mm × 100 mm, 1.7 μm; Waters, which was maintained at 45 °C. The injection volume was 4 μL and the flow rate was 300 μL /min. The mobile phase consisted of 15 mM ammonium acetate in water (A) and acetonitrile (B). The initial gradient condition was 90% B, which was decreased to 40% gradually over 18 min, to 90% after 18.1 min, and then maintained at 90% B from 18.1 to 23 min.

The MS system was operated using electrospray ionization (ESI) in negative mode. The optimum conditions were source temperature, 450 °C; ion Source Gas1 (Gas1), 45; Ion Source Gas2 (Gas2), 45; Curtain gas (CUR), 30; ion sapary voltage floating (ISVF), 4500 V. The MRM transitions, retention times, and conditions are shown in Supplementary Data 18. Data were processed using Multiquant software.

### DNA extraction for metagenome profiling

DNA was extracted from frozen fecal samples using E.Z.N.A.^®^ DNA Kit (Omega Biotek, Norcross, GA, U.S.) according to the manufacturer’s protocols. The DNA concentration and purity was quantified with TBS-380 and NanoDrop2000, respectively. DNA quality was assessed with the 1% agarose gel electrophoresis system. After final precipitation, the DNA samples were resuspended in TE buffer and stored at −80 °C for further analysis.

### Whole-genome shotgun sequencing

DNA was first fragmented to an average size of about 300 bp using Covaris M220 (Gene Company Limited, China). And Paired-end library was constructed by using the TruSeqTM DNA Sample Prep Kit (Illumina, San Diego, CA, USA). Adapters containing the full complement of sequencing primer hybridization sites were ligated to the Blunt-end fragments. Whole-genome shotgun sequencing of fecal samples was carried out on the Illumina HiSeq4000 platform (Illumina Inc., San Diego, CA, USA) with HiSeq 3000/4000PE Cluster Kit and HiSeq 3000/4000 SBS Kits (www.illumina.com).

### Sequence preprocessing and taxonomic and functional profiling

Fecal metagenomic shotgun sequences were trimmed for adapters and low-quality bases using trimmomatic, discarding reads of lengths less than 50 base pairs and reads of average quality less than 20. Filtered reads were then aligned to the human genome (hg 19) using bowtie2. And those mapped were considered unwanted human reads and were discarded. FastQC was then used for quality control prior to downstream analysis.

To identify reads belonging to rats, we first downloaded the reference genome of rat from NCBI. And then, we align the obtained metagenomic sequence reads to the rat reference genome using Bowtie2 (version bowtie2-2.1.0). By doing this, we identified and deleted the reads belonging to rat. MetaPhlAn2^46^ was applied to quantitatively profile the taxonomic composition of the metagenome of all metagenomic samples, whereas HUMANn2^47^ was conducted with the UniRef90 database for functional analysis. The HUMAnN2 algorithm generated pathway abundance, gene-family abundance and pathway coverage. Linear discriminant analysis (LDA) effect size (LEfSe), using a factorial Kruskal–Wallis and LDA test, was applied to identify significant differences in bacterial groups.

### Functional bacteria analysis

Through extensive searching, exploring, and reading literature, we obtained the potential functional role of every bacteria identified. Detailed information about the functions/characteristics of the species searched from the literature is listed in Supplementary Data 19. The total abundance of the functional bacteria (oxygen utilization, anti-inflammation bacteria, pro-inflammation bacteria, neurotransmitter-producing bacteria) was calculated.

### PCAtoTree

For metabolomics data with different metabolic states, it will be difficult to evaluate the similarity or difference between the respective data point clusters in PCA score plots^62^. Here, PCAtoTree^28^ was applied for improved quantitative analysis in score plots and for better visualization. Employing both nonparametric and parametric hypothesis testing, it can quantitatively describe significant differences between different groups through dendrograms and bootstrapping techniques.

### Multiblock

Metabolites detected in LC/MS and GC/MS are different and can complement each other. To get a comprehensive view of the metabolites, it is better to fuse LG/MS and GC/MS datasets of the same samples. Multiblock methods were used here to merge datasets from different platforms by extracting latent variables from each block, which was considered information carriers^25^. Multiblock-PCA can be applied for visualizing the overall tendency of the fusing data. And multiblock-PLS (MBPLS), a kind of regression model, can be used for variable selection using the regression coefficients (the b values). These multiblock methods were performed using the ade4 R package.

### Differential analysis

The linear discriminant analysis (LDA) effect size (LEfSe) analysis was performed to compare the relative abundance of the different features between groups. LDA score threshold of >2.0 and a 0.05 alpha value for the factorial Kruskal–Wallis test was applied to select different bacteria.

A series of strict filtering criteria were applied to select significant different features for gut metabolites, KO and pathway abundance data. The VIP (Variable Importance in Projection) value, representing the importance of selected metabolites in the discriminating OPLS-DA model, was first used to filter variables (VIP value >1). Then the dataset was further screened using Wilcoxon test *p* value <0.05 and absolute Log2 fold change (Log2FC) >1. The volcano Plot was used to simultaneously display the statistical significance (*P* value) versus the magnitude of change (Log2 fold change). We could get a quick visual identification of features with large fold changes that are also statistically significant, which may be the most biologically significant features.

### Microbial driver species analysis

Microbial driver species analysis was a pipeline referred to the methods explained in literature^49^, which can combine three data dimensions (microbial species, microbial functions and clinical phenotypes). Using a so-called leave-one-out analysis, this method can identify species which contributed particularly to the observed linkage between clinical phenotype and metagenomic functional potential. And the identified species was considered a driver species, which may have a great influence on the functions of the microbiome. The detailed analysis framework has been reported as source R code at the accompanying Git repository (https://bitbucket.org/hellekp/clinical-micro-meta-integration)

### MIMOSA

A novel comprehensive framework, the Model-based Integration of Metabolite Observations and Species Abundances 2 (MIMOSA2)^56^, is used for metabolic model-based evaluation of paired microbiome-metabolome datasets to support mechanistic interpretation and hypothesis generation. MIMOSA2 first constructs community metabolic models, and then assesses whether metabolite measurements are consistent with estimated community metabolic potential, followed by identifying specific taxa and reactions that can explain metabolite variation. The MIMOSA2 web application is available at http://elbo-spice.cs.tau.ac.il/shiny/MIMOSA2shiny/.

### mixOmics

MixOmics^26^ is an R package dedicated to integrate multivariate “omics biological data. By adopting a biology approach, the toolkit provides a wide range of methods focused on data exploration, dimension reduction and visualization. Among which, the toolkit can provide a supervised framework” analyses (Data Integration Analysis for Biomarker discovery using Latent cOmponents, or DIABLO for short)^27^ to probe relationships between different tissues by identifying a multi-tissue signature composed of highly correlated features across different tissues and which can discriminate the outcome of interest. The packages can be obtained on the website http://mixomics.org.

### Statistics and reproducibility

All the data analysis and the drawing of the diagrams were implemented in the R package (version 4.0.4, http://www.r-project.org). Mann–Whitney *U*-test was used to select different features. *P* < 0.05 was considered statistically significant.

For the global metabolite profiling, 29 rats were used (*n* = 7 in Sham, *n* = 6 in IIR0h, *n* = 9 in IIR6h, *n* = 7 in IIR72h). For the metagenomic analysis of gut contents, 35 rats were used (*n* = 10 in Sham, *n* = 15 in IIR0h, *n* = 10 in IIR6h). And for detection of the bowel barrier damage-related biomarkers and immunohistochemical analysis, three biologically independent animals were used.

### Reporting summary

Further information on research design is available in the Nature Research Reporting Summary linked to this article.

## Supplementary information

Supplementary Information

Description of Additional Supplementary Files

Supplementary Data 1

Supplementary Data 2

Supplementary Data 3

Supplementary Data 4

Supplementary Data 5

Supplementary Data 6

Supplementary Data 7

Supplementary Data 8

Supplementary Data 9

Supplementary Data 10

Supplementary Data 11

Supplementary Data 12

Supplementary Data 13

Supplementary Data 14

Supplementary Data 15

Supplementary Data 16

Supplementary Data 17

Supplementary Data 18

Supplementary Data 19

Supplementary Data 20

Supplementary Data 21

Supplementary Data 22

Supplementary Data 23

Supplementary Data 24

Reporting Summary

## Data Availability

The metagenomics data have been deposited into the NCBI database under the BioProject accession number PRJNA669162. The authors declare that all other data supporting the findings of the study are available in the paper and online supplementary materials.

## Code availability

The in-house codes for all the analysis, figures, and tables in the manuscript can be achieved upon reasonable request.
