# Supplementary material for: Integrated multi-omics reveal important roles of gut contents in intestinal ischemia–reperfusion induced injuries in rats
Source: Commun Biol. 2022 Sep 9;5:938. doi: 10.1038/s42003-022-03887-8 (PMC9463172; doi:10.1038/s42003-022-03887-8)
Supplement: Supplementary file 1 — Supplementary Information [file 42003_2022_3887_MOESM1_ESM.pdf]

Supplementary Materials

Integrated multi-omics reveal important roles of gut contents in intestinal ischemia–reperfusion induced injuries in rats

Die Dai[1], Fandie Dai[2], Jingchao Chen[1], Menglu Jin[3], Mingyue Li[4], Desheng Hu[4], Zhi Liu[5], Zunjian Zhang[6,7,8], Fengguo Xu[6,7,8*], Wei-Hua Chen[1,3*]

[1]Key Laboratory of Molecular Biophysics of the Ministry of Education, Hubei Key Laboratory of Bioinformatics and Molecular-Imaging, Center for Artificial Intelligence Biology, Department of Bioinformatics and Systems Biology, College of Life Science and Technology, Huazhong University of Science and Technology, Wuhan, China

[2]Applied Psychology Institution, China University of Geosciences, Wuhan, China

[3]College of Life Science, Henan Normal University, Xinxiang, China

[4]Department of Integrated Traditional Chinese and Western Medicine, Union Hospital, Tongji Medical College, Huazhong University of Science and Technology, Wuhan, China

[5]Department of Biotechnology, Huazhong University of Science and Technology, College of Life Sciences and Technology, Wuhan, China

[6]Key Laboratory of Drug Quality Control and Pharmacovigilance, Ministry of Education, China Pharmaceutical University, Nanjing, China

[7]Jiangsu Key Laboratory of Drug Screening, China Pharmaceutical University, Nanjing, China

[8]State Key Laboratory of Natural Medicine, China Pharmaceutical University, Nanjing, China

* correspondence authors

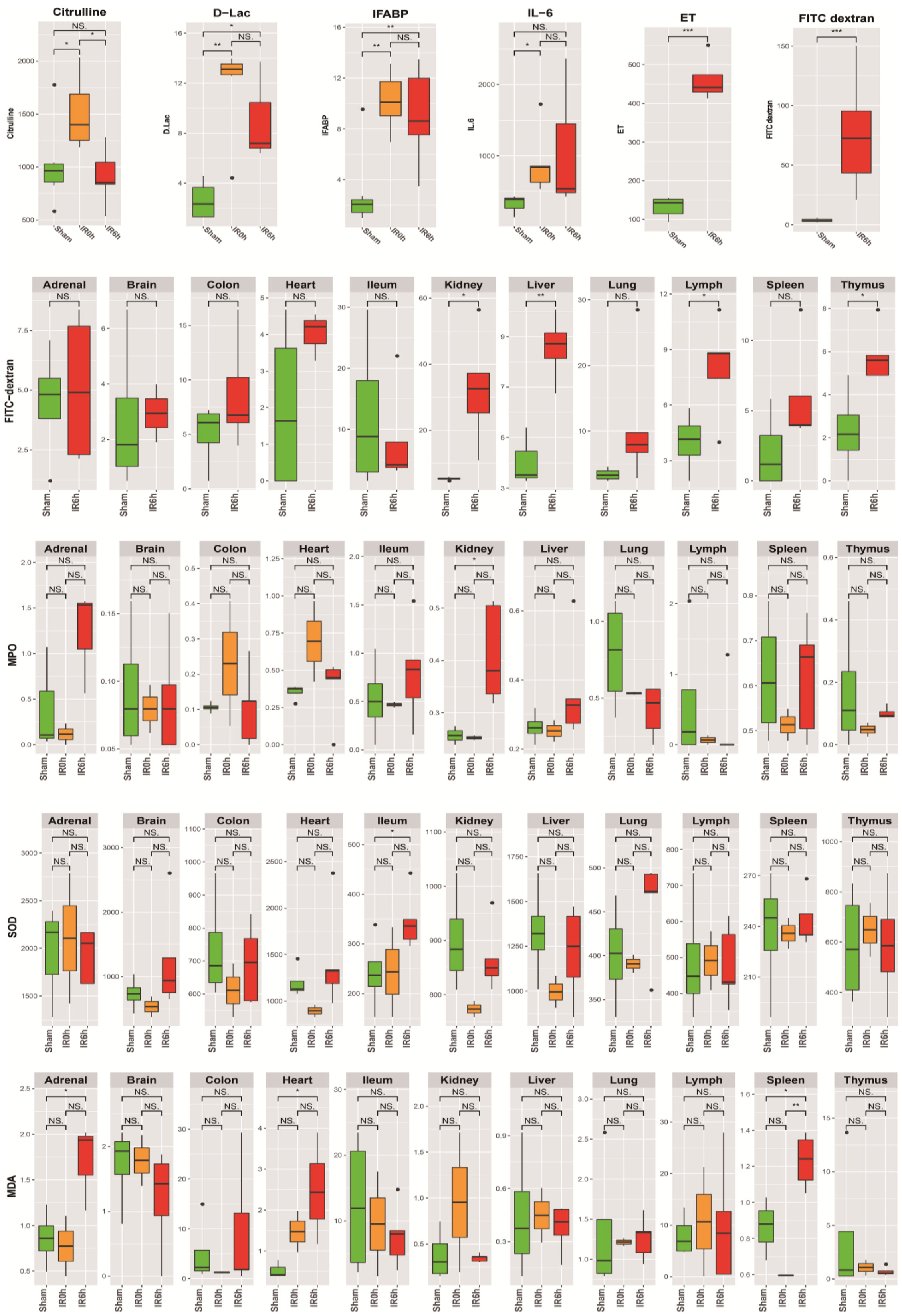

Supplementary Figure 1 Concentration alteration of some oxidative stress damage related indexs (MPO, SOD, MDA) and some bowel barrier damage related biochemical indexes: Fluorescein isothiocyanate (FITC)-dextrans, citrulline, D-lactic acid (D-Lac), Intestinal-type fatty acid-binding protein (IFABP), endotoxin (ET) and IL-6. MPO, myeloperoxidase; SOD, Superoxide dismutase; MDA, Malondialdehyde). Between group comparisons were performed using the Wilcox test; *, $p < 0.05$; **, $p < 0.01$; ***, $p < 0.001$; NS, not significant. The box represents the median, 25th, and 75th percentiles and the error bars indicate the 5th and 95th percentiles.

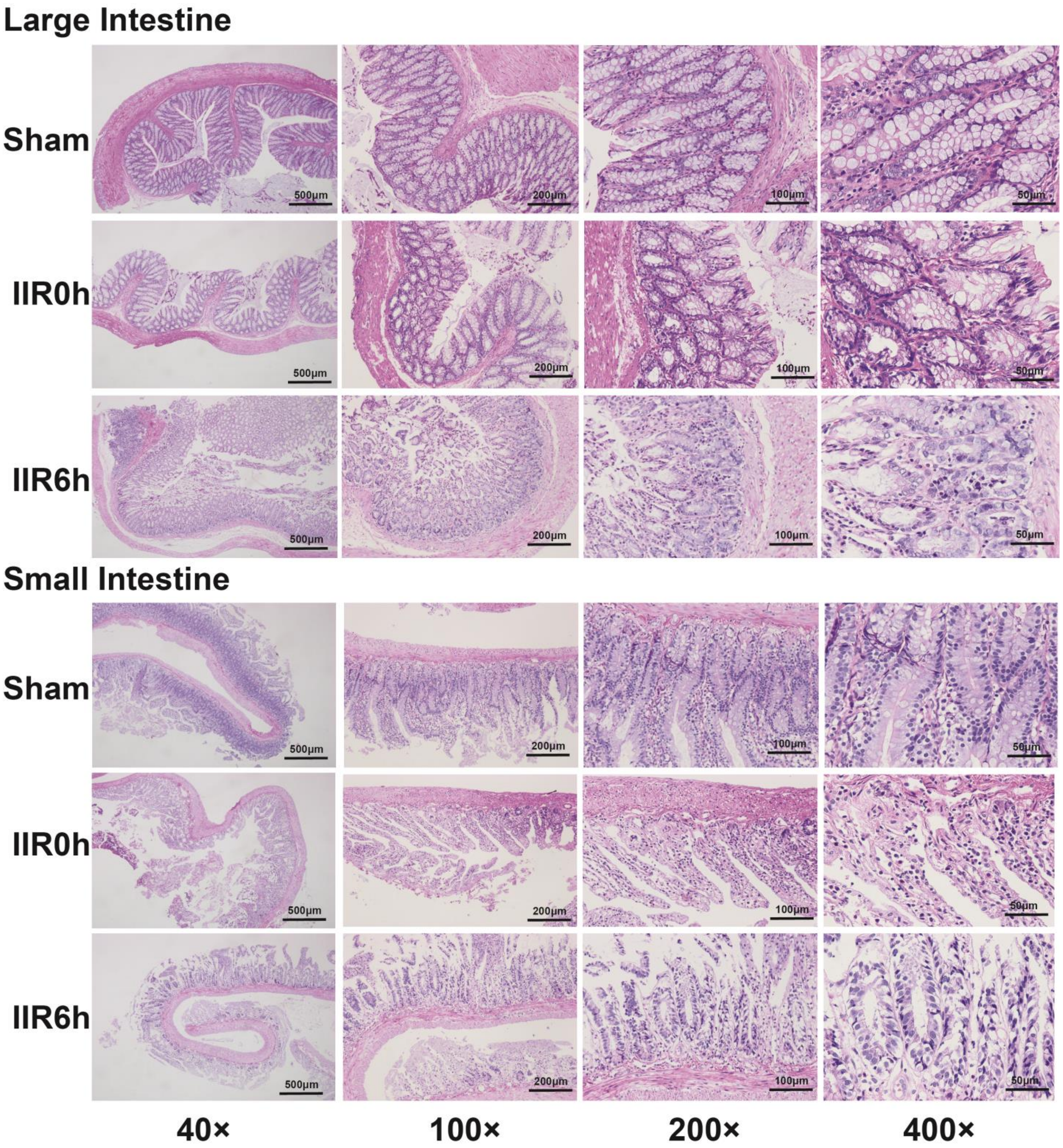

Supplementary Figure 2 HE staining of intestine during IIR.

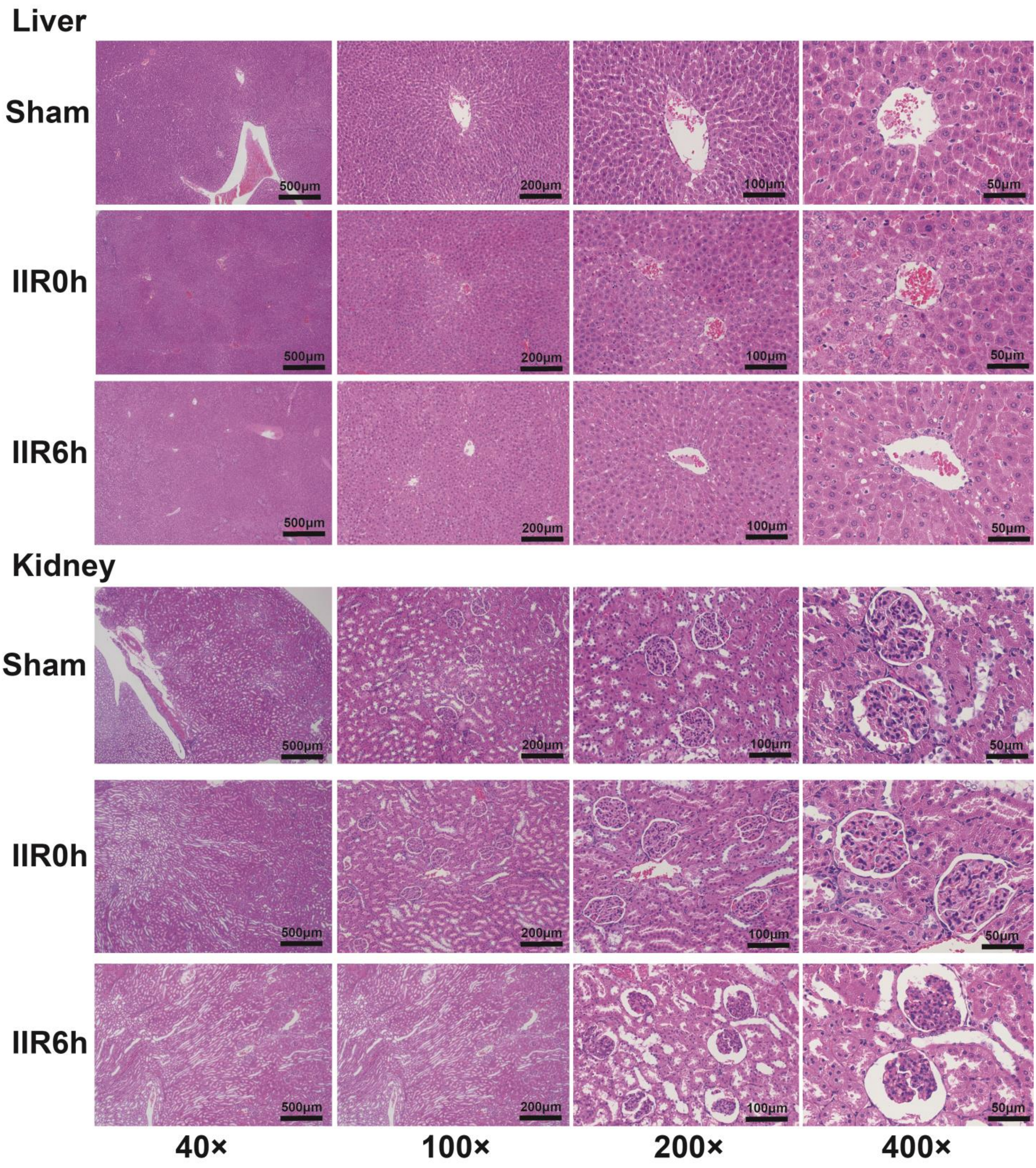

Supplementary Figure 3 HE staining of liver and kidney during IIR.

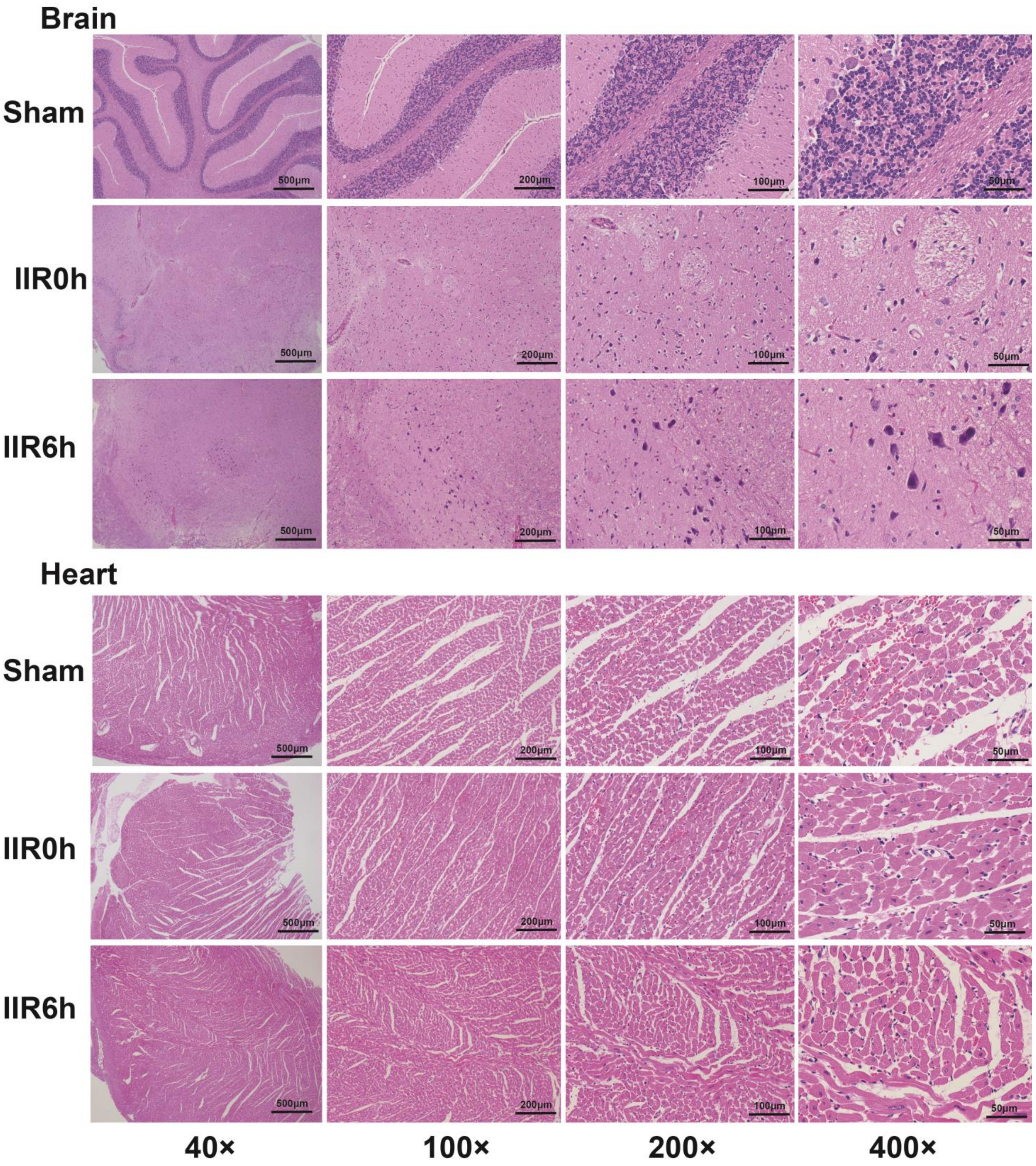

Supplementary Figure 4 HE staining of brain and heart during IIR.

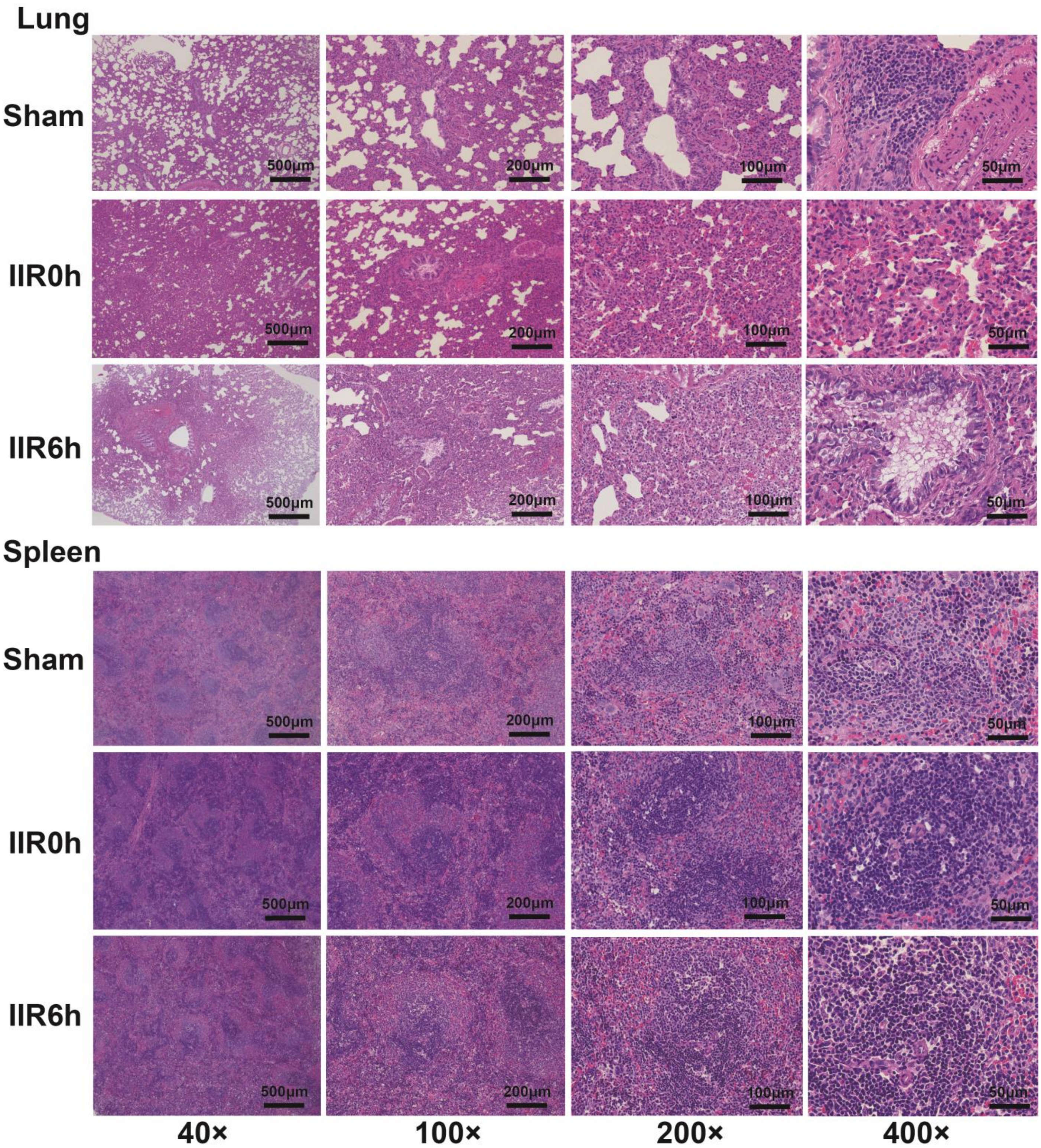

Supplementary Figure 5 HE staining of lung and spleen during IIR.

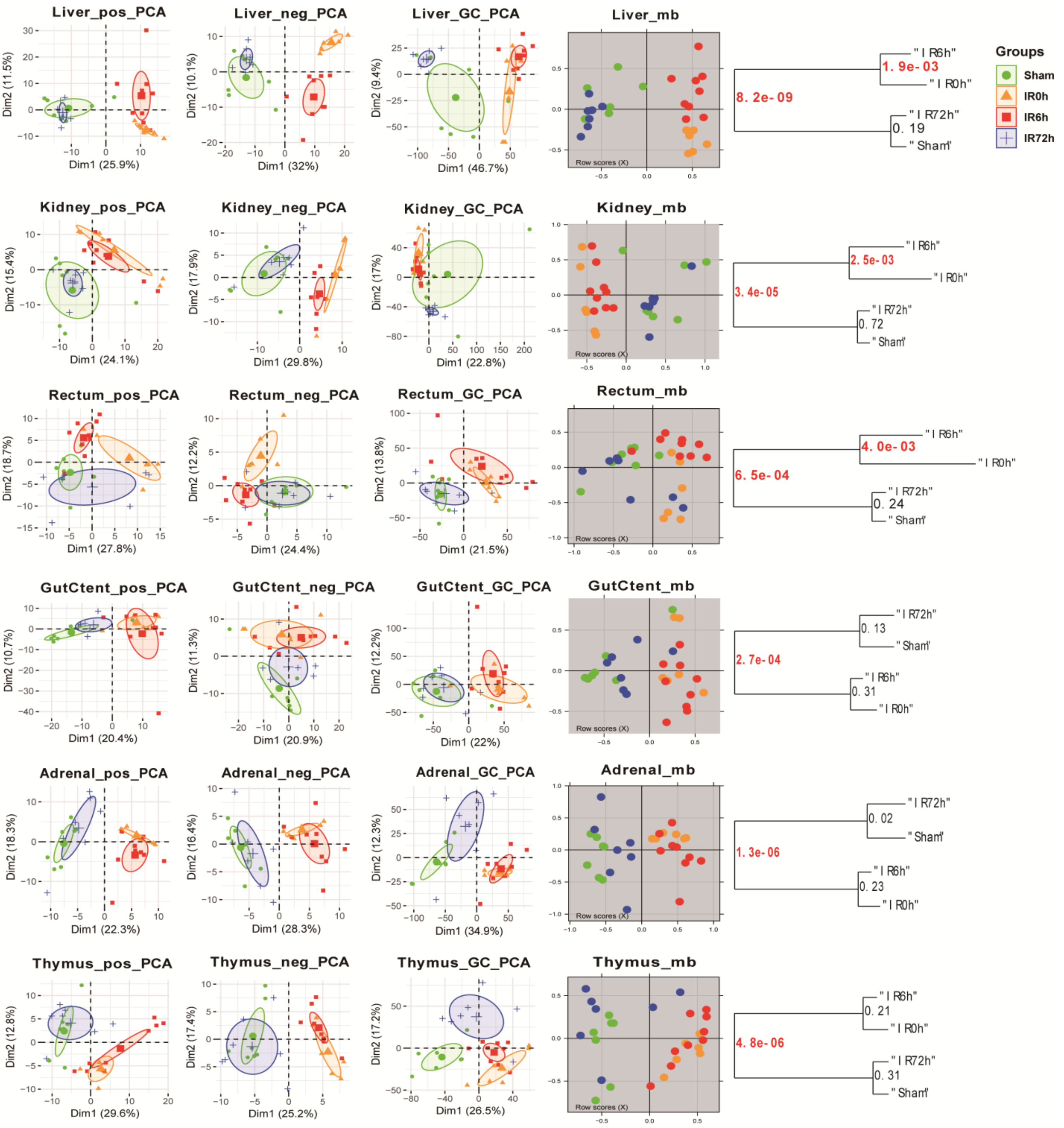

Supplementary Figure 6 Global metabolomics profiling using mass spectrometry revealed significantly influenced tissues by IIR (represents in each row: liver, kidney, rectum, gut content, adrenal and thymus). The first three columns are the principal component analysis (PCA) score plots derived from the LC/MS (ESI+), LC/MS (ESI-) and GC/MS metabolite data respectively. And the fourth column illustrates the multiblock principal component analysis of the data fusion of LC/MS and GC/MS. Plots in the fifth column are metabolic tree diagrams corresponding to the multiblock PCA score plots using Mahalanobis distances, with p-values for the null hypothesis shown at each branch. The first two principal components (Dim1 and Dim2) were estimated as sufficiently well represented by the principal plan generated by each PCA. The value of $p < 0.01$ was considered to be significant different, which were marked in red. Green symbols correspond to Sham groups, yellow symbols to IR0h groups, red ones to IR6h groups and blue ones to IR72h groups. Pos mode: liquid chromatography-mass spectrometry (ESI+); Neg mode: liquid chromatography-mass spectrometry (ESI-); GC Mode: gas chromatography-mass spectrometry.

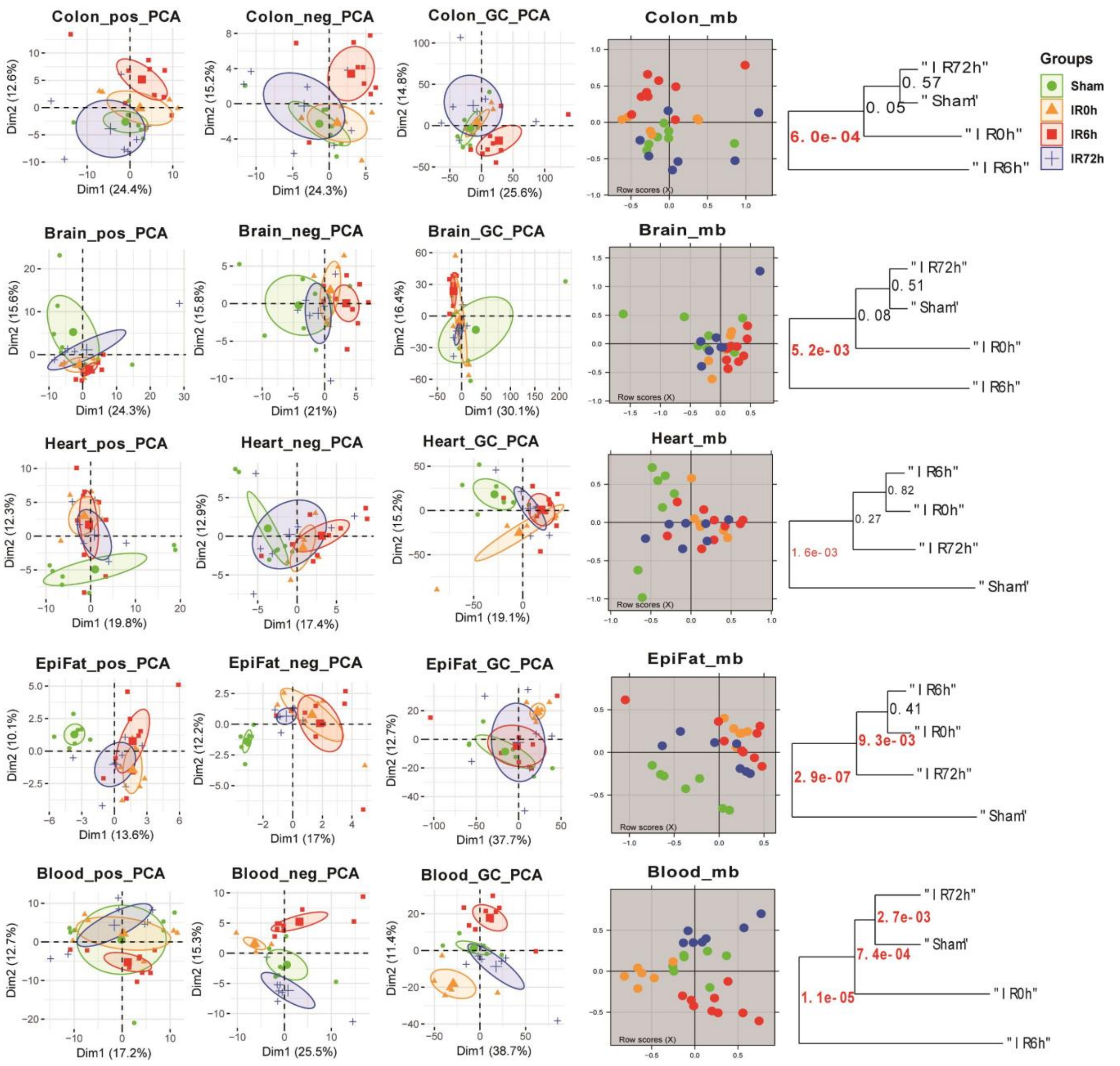

Supplementary Figures 7 Global metabolomics profiling using mass spectrometry revealed influenced tissues by IIR (represents in each row: colon, brain, heart, epididymal fat and blood). The first three columns are the principal component analysis (PCA) score plots derived from the LC/MS (ESI+), LC/MS (ESI-) and GC/MS metabolite data respectively. And the fourth column illustrates the multiblock principal component analysis of the data fusion of LC/MS and GC/MS. Plots in the fifth column are metabolic tree diagrams corresponding to the multiblock PCA score plots using Mahalanobis distances, with p-values for the null hypothesis shown at each branch. The first two principal components (Dim1 and Dim2) were estimated as sufficiently well represented by the principal plan generated by each PCA. The value of $p < 0.01$ was considered to be significant different, which were marked in red. Pos mode: liquid chromatography-mass spectrometry (ESI+); Neg mode: liquid chromatography-mass spectrometry (ESI-); GC Mode: gas chromatography-mass spectrometry.Hear

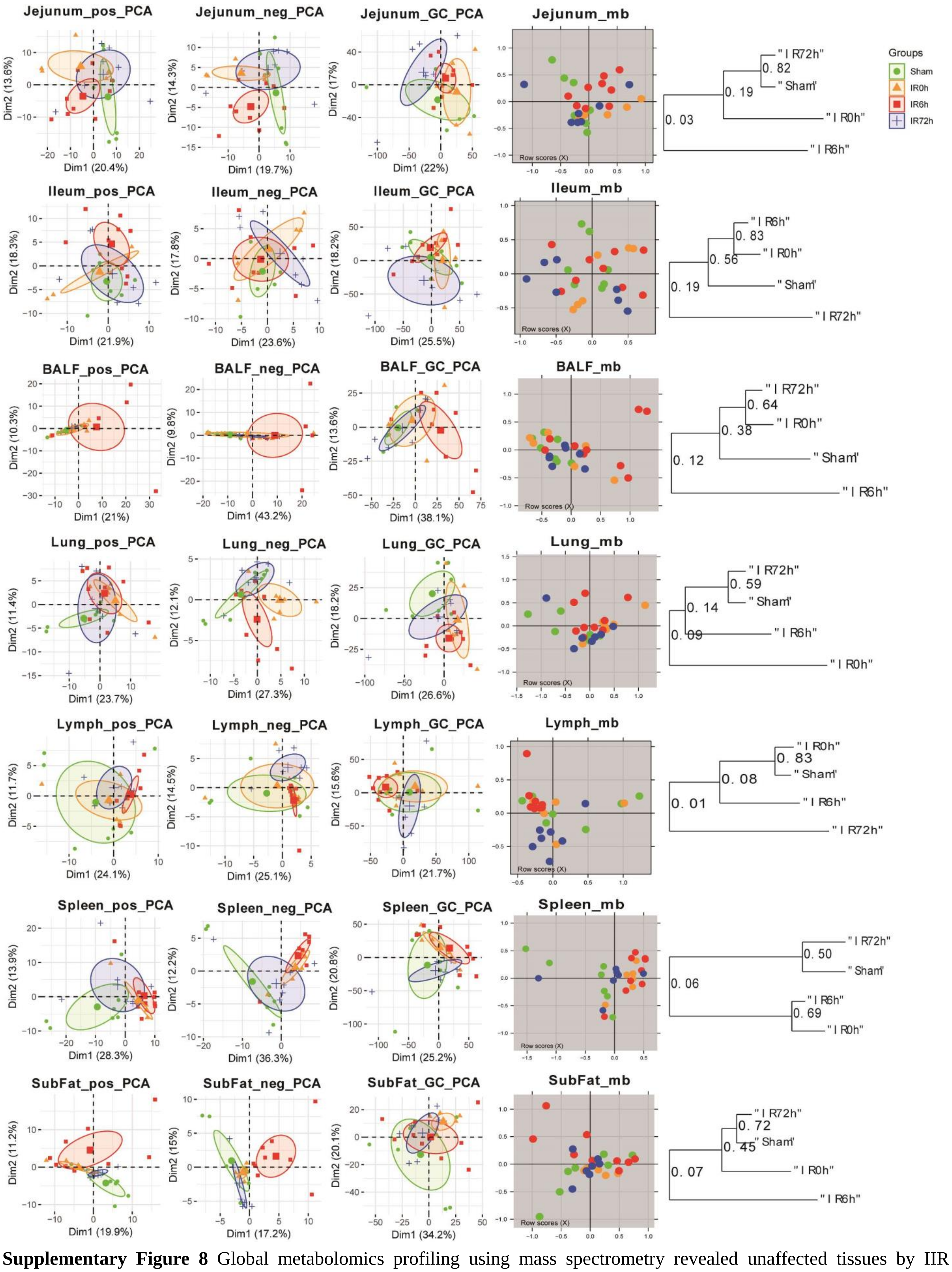

Supplementary Figure 8 Global metabolomics profiling using mass spectrometry revealed unaffected tissues by IIR (represents in each row: jejunum, ileum, BALF, lung, lymph, spleen and subcutaneous fat). The first three columns are the principal component analysis (PCA) score plots derived from the LC/MS (ESI+), LC/MS (ESI-) and GC/MS metabolite data respectively. And the fourth column illustrates the multiblock principal component analysis of the data fusion of LC/MS and GC/MS. Plots in the fifth column are metabolic tree diagrams corresponding to the multiblock PCA score plots

using Mahalanobis distances, with p-values for the null hypothesis shown at each branch. The first two principal components (Dim1 and Dim2) were estimated as sufficiently well represented by the principal plan generated by each PCA. The value of $p < 0.01$ was considered to be significant different, which were marked in red. Pos mode: liquid chromatography-mass spectrometry (ESI+); Neg mode: liquid chromatography-mass spectrometry (ESI-); GC Mode: gas chromatography-mass spectrometry. BALF, bronchoalveolar lavage fluid.

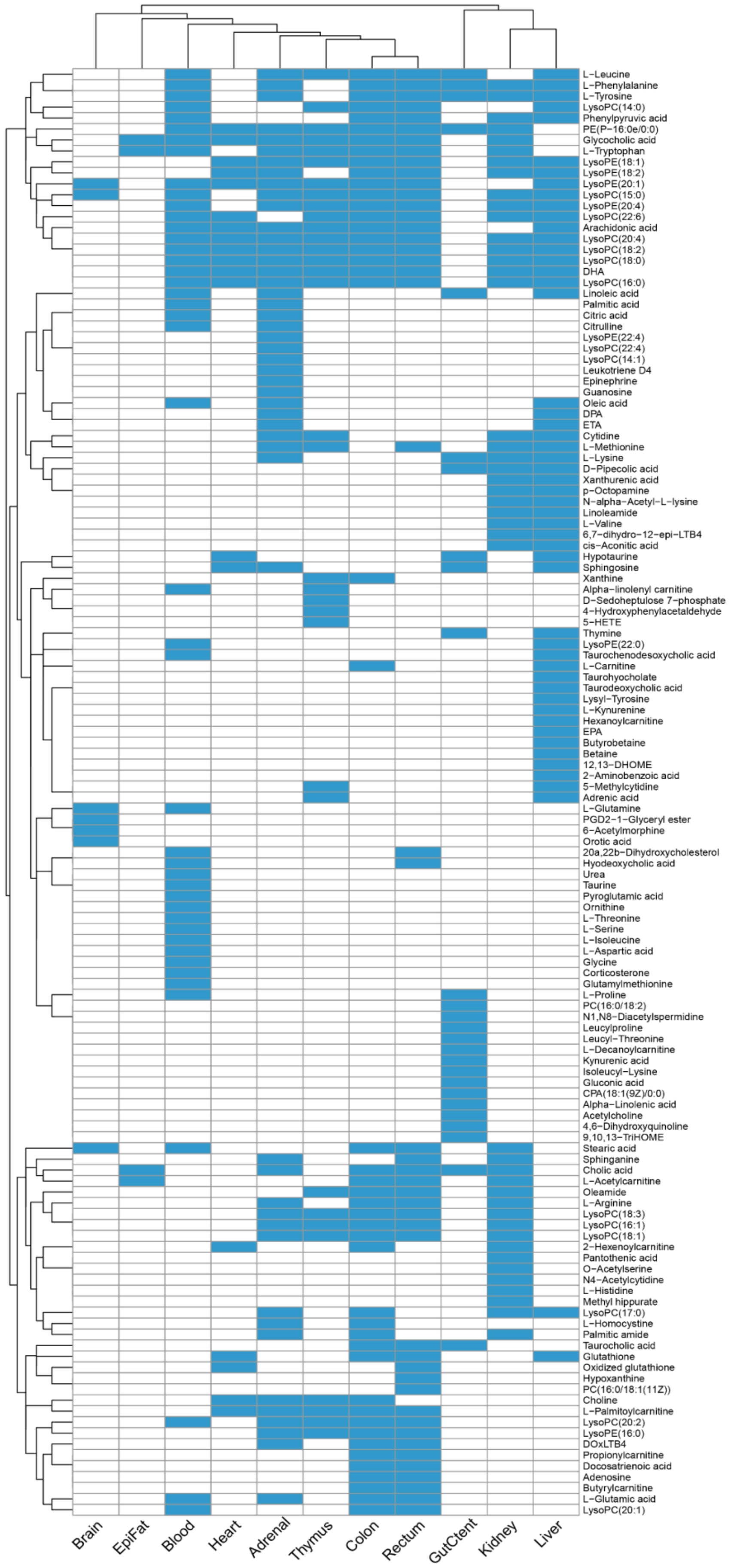

Supplementary Figure 9 Identified significantly changed metabolites in each tissue. Each tissue has its specific metabolites. The blue block represents the metabolite can be detected in the tissue; the white block represents the metabolite cannot be detected in the tissue.

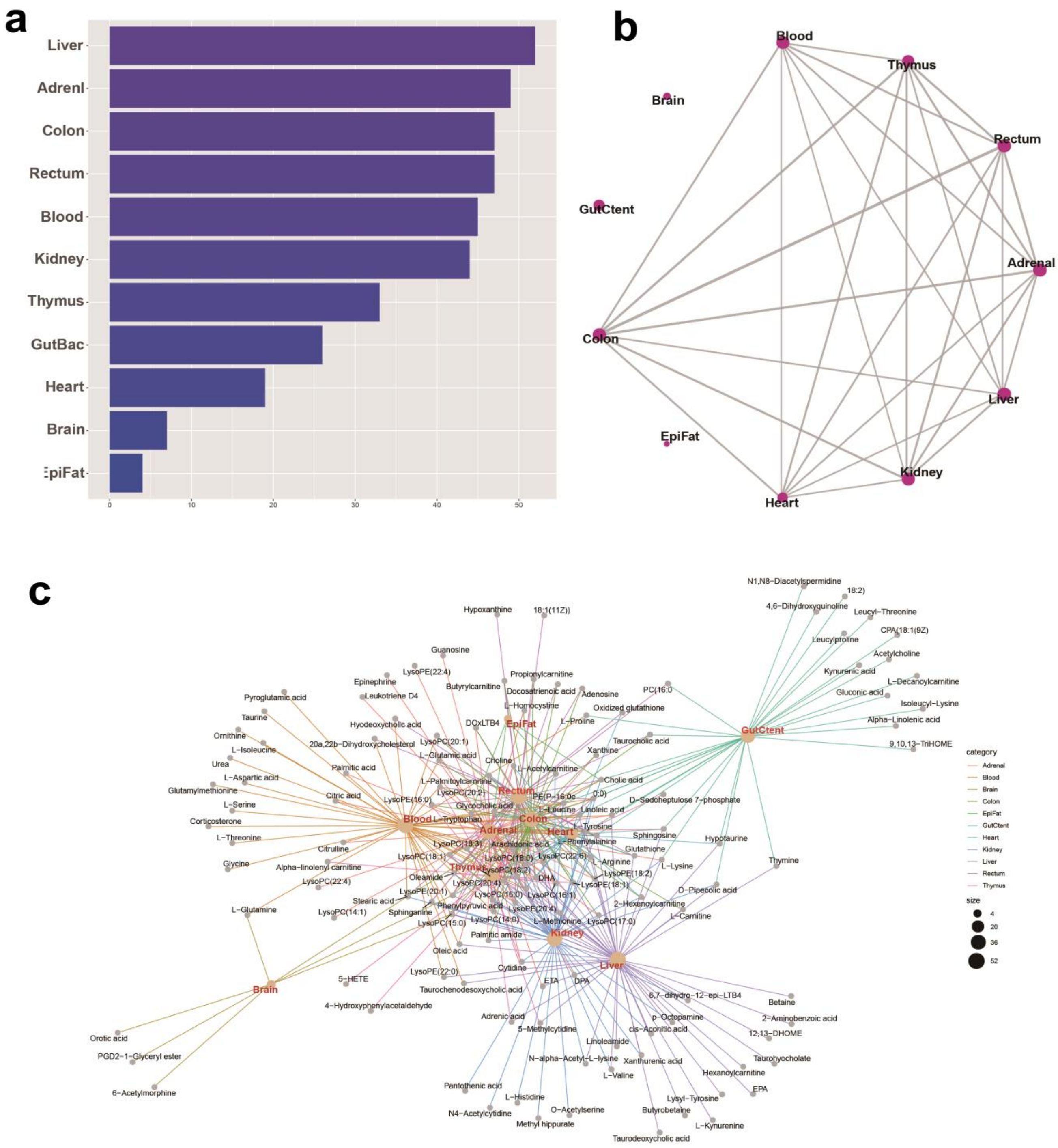

Supplementary Figure 10 Characteristics of metabolite profile for each tissue. (a) Number of identified significantly different metabolites in each tissue. Most metabolites identified in liver, adrenal comes second, and epididymal fat has the least. GutBac, gut content; EpiFat, epididymal fat. **(b)** Links between tissues/blood/gut content based on the shared metabolites in each tissue. The class distribution of metabolites was similar across tissues except brain, gut content and epididymal fat. **(c)** Links between tissues based on the identified shared metabolites.

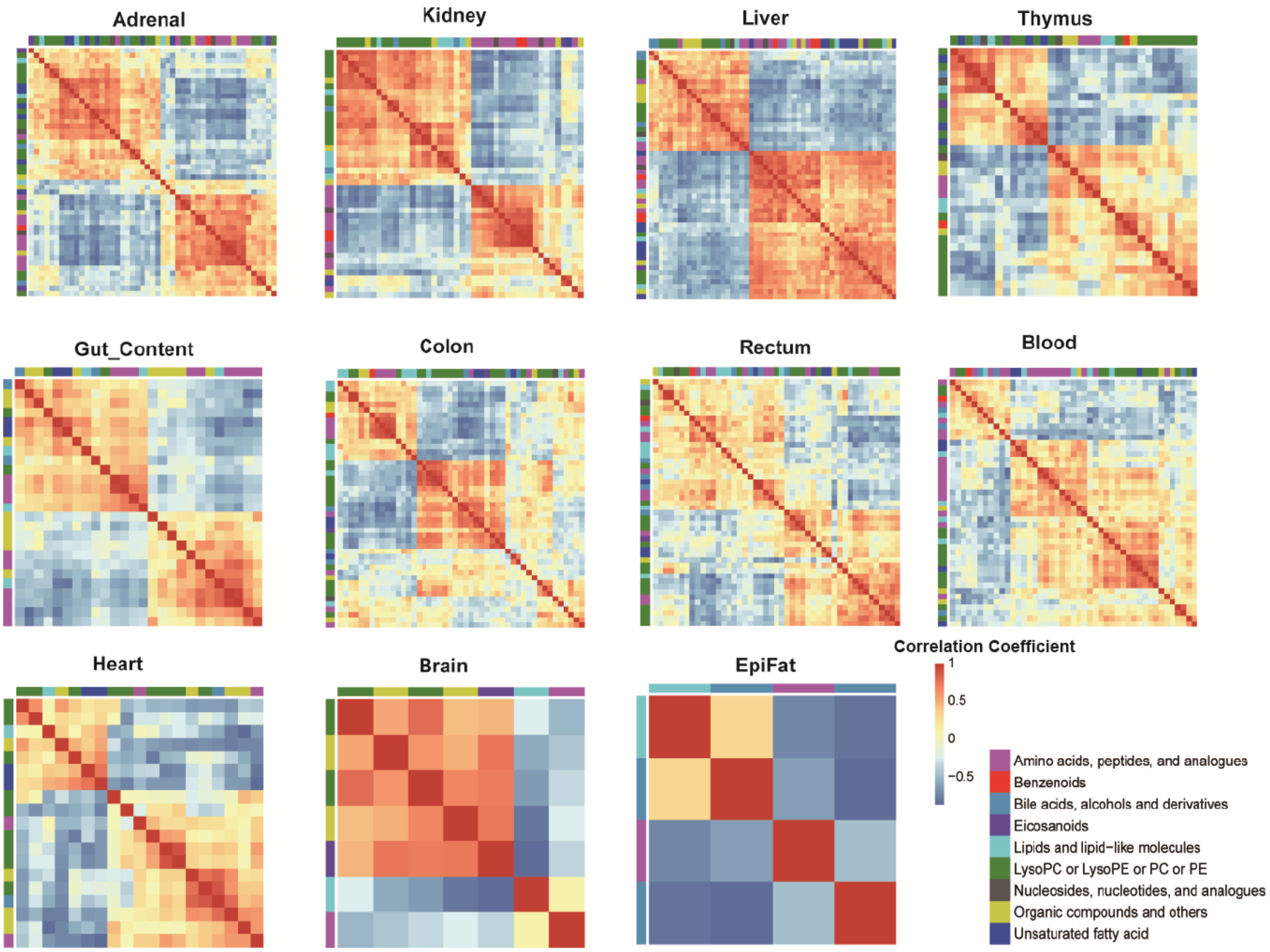

Supplementary Figure 11 Inter-tissue correlations of metabolites. The modes were different between tissues. Some could be divided into two parts (adrenal, kidney, liver, thymus, gut content, rectum and heart), whereas others didn't show this phenomena. Blue square indicates a reduction, and red ones represent an increase. The units for the heatmap color key represent the correlation coefficient, the value of which is between -1 and 1.

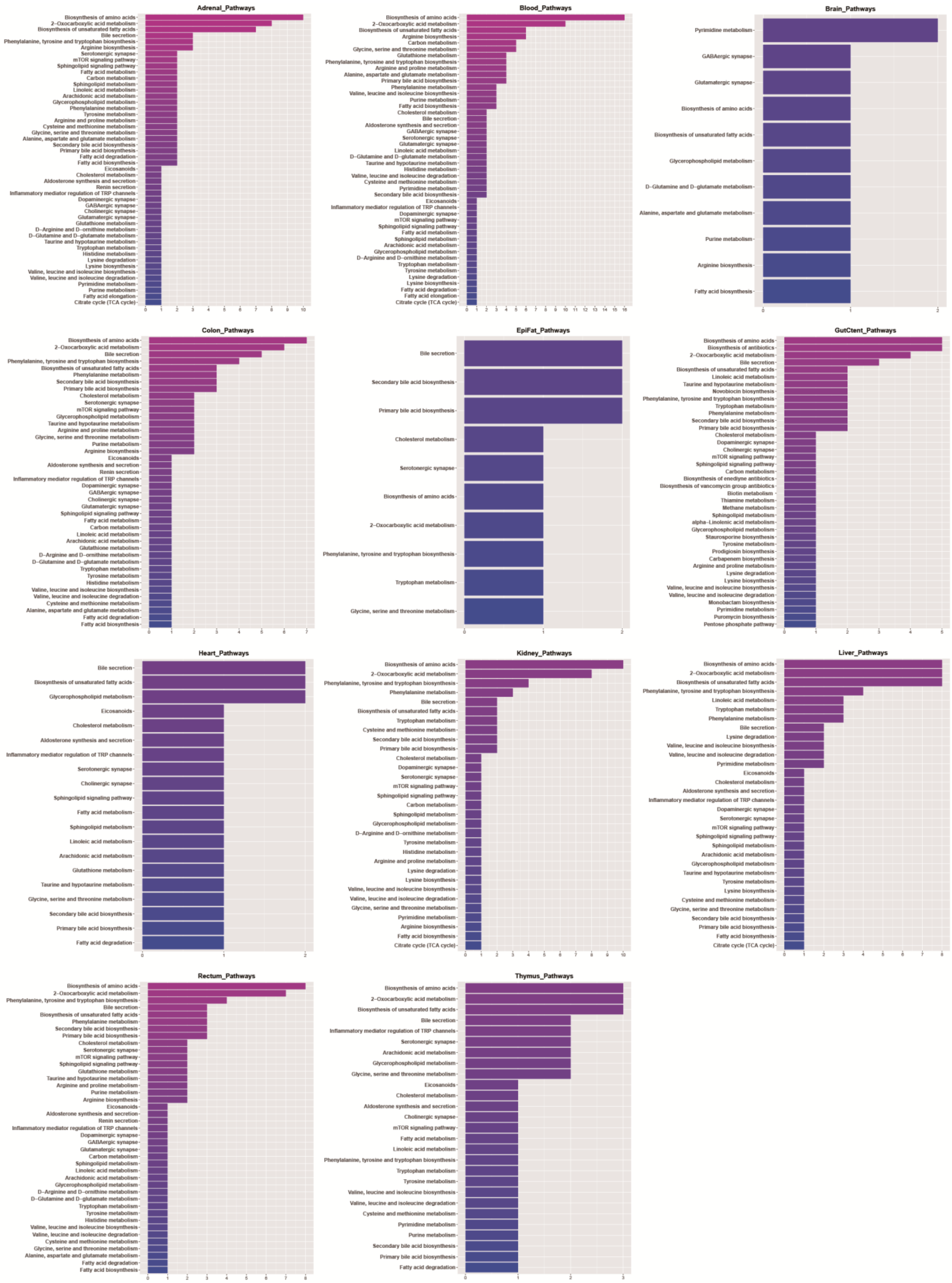

Supplementary Figure 12 Bar plots showing important pathways in each tissue by pathway enrichment analysis.

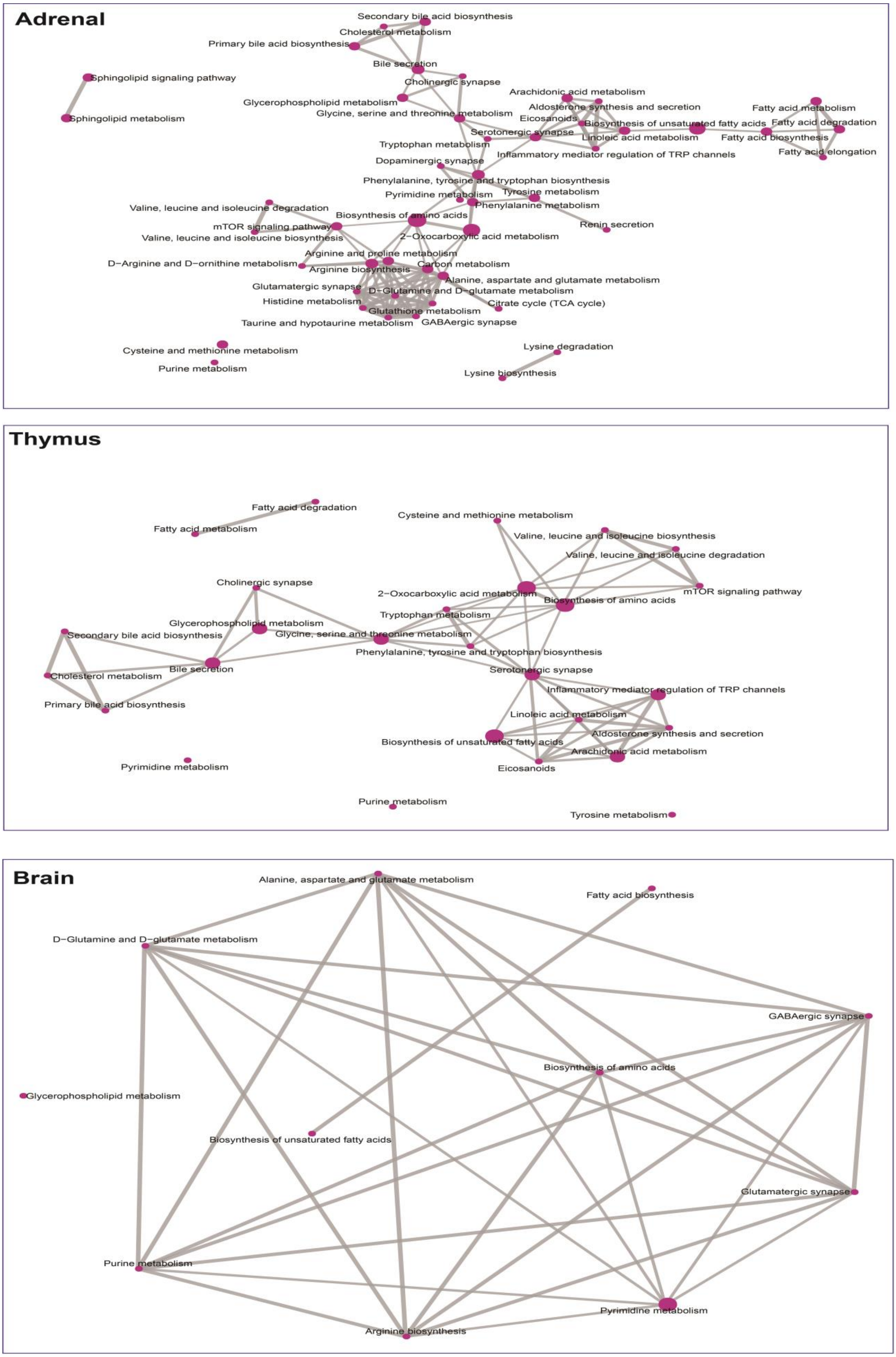

Supplementary Figure 13 Network analysis using the identified metabolites based on pathway enrichment analysis, showing the important potential functions in each tissue.

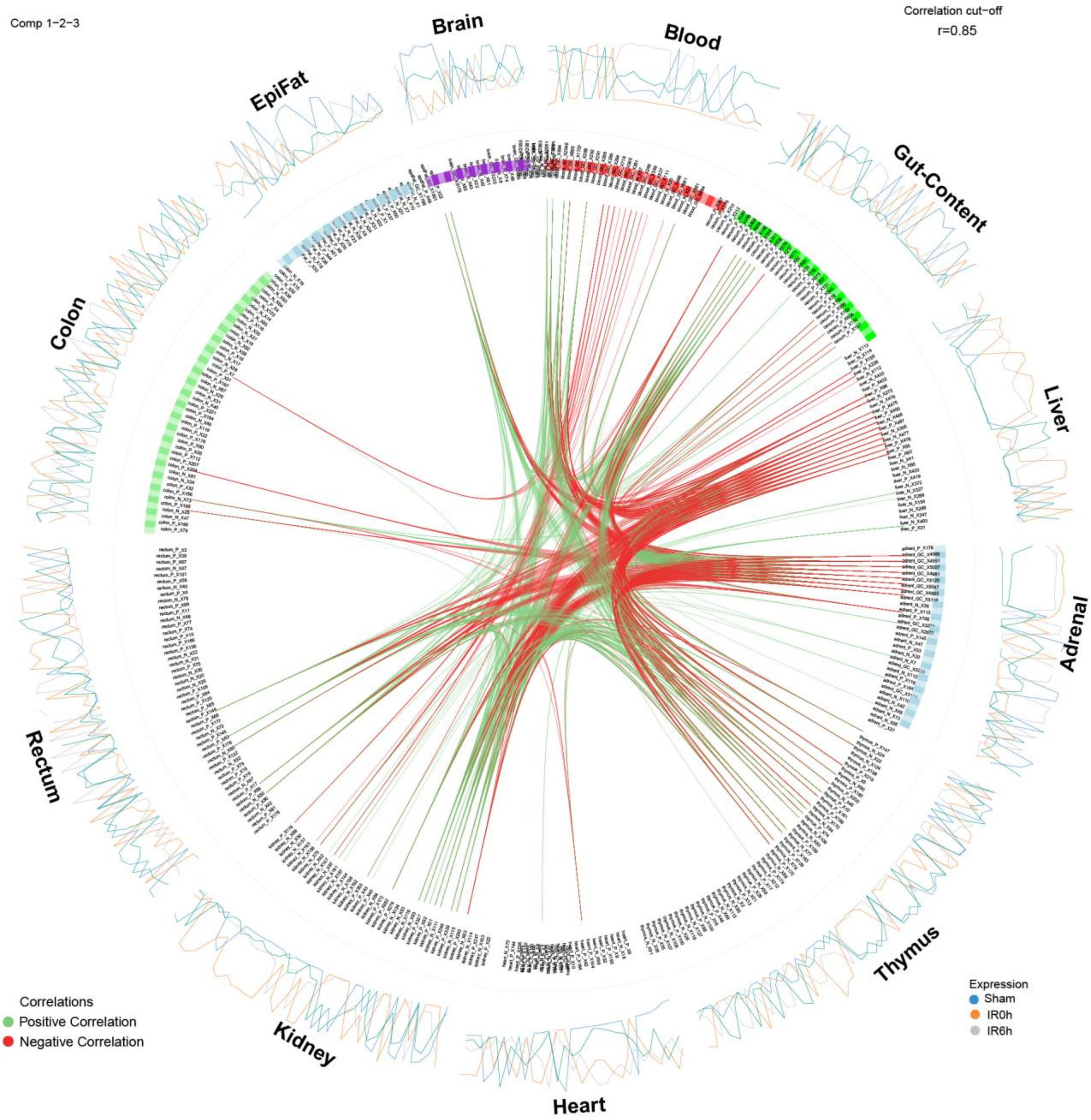

Supplementary Figure 14 Relevance network based on the similarity matrix was built to visualize the correlations between metabolites of different tissues. The threshold which represents the correlation coefficient was set to 0.85.

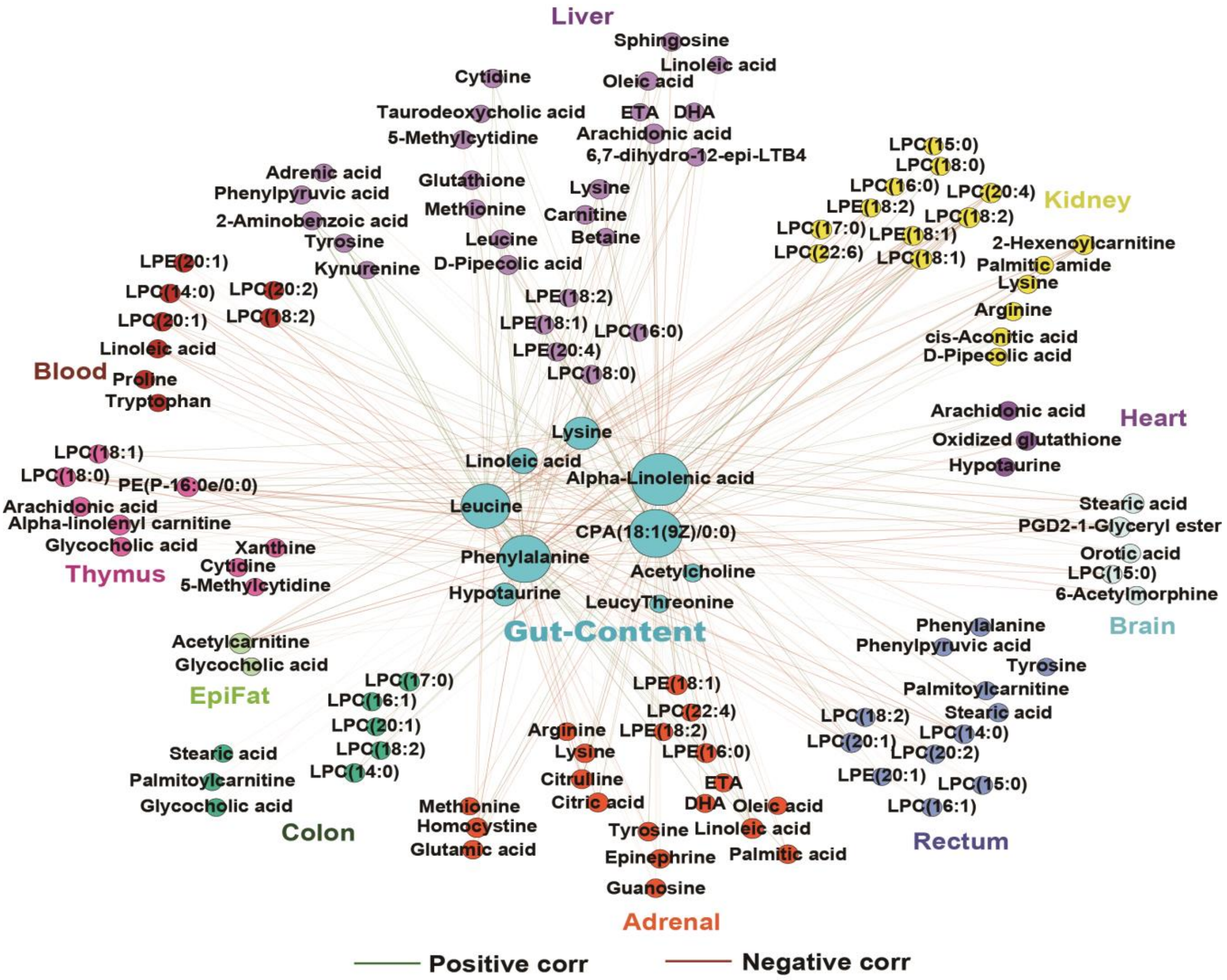

Supplementary Figure 15 Correlations between gut content and all the other tissues at metabolite level. The edges designate the correlation coefficients above 0.7.

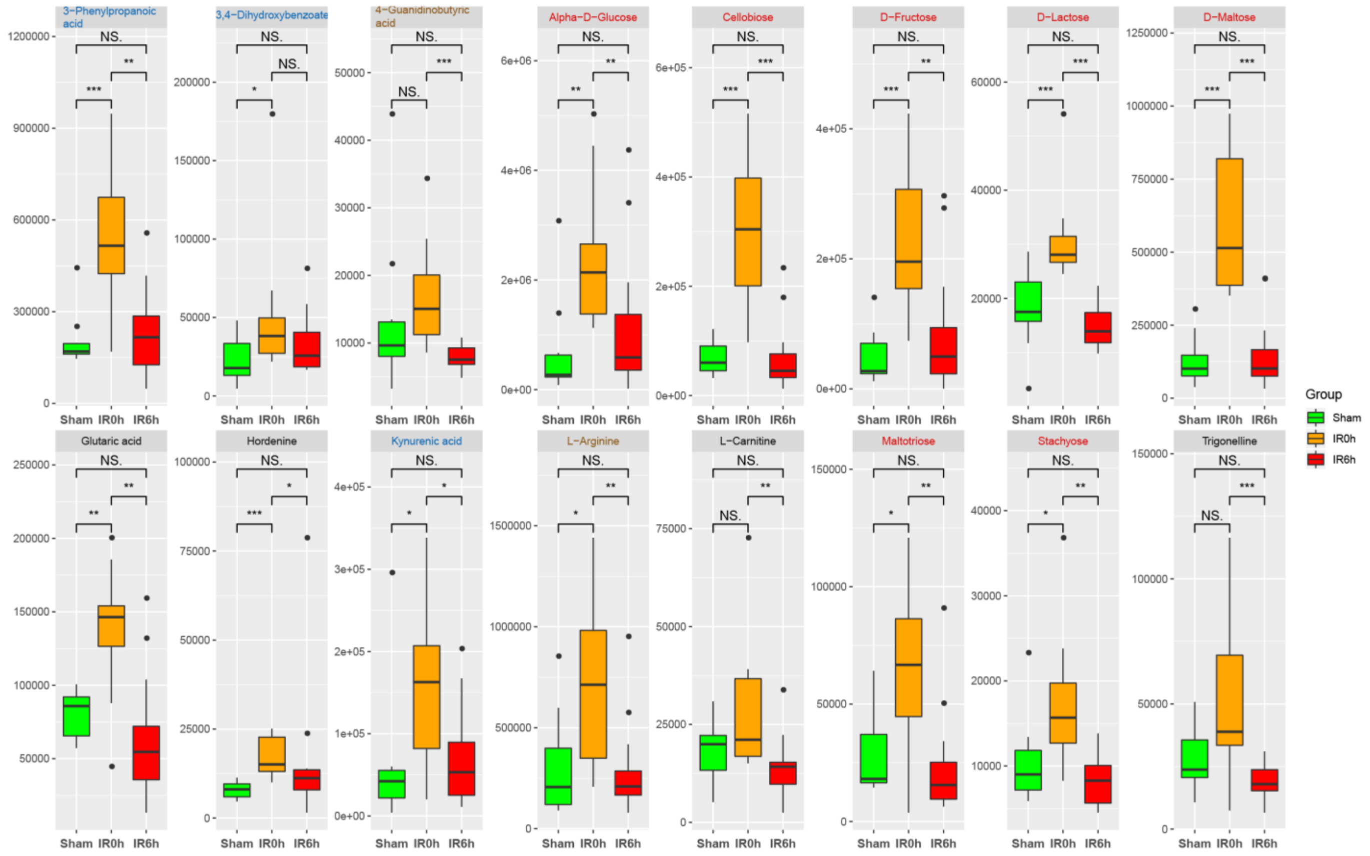

Supplementary Figure 16 Alteration of the metabolome in gut content during IIR. Metabolites which first rose at IIR0h and then dropped to normal level at IIR6h. Between group comparisons were performed using the Wilcox test; *, $p < 0.05$; **, $p < 0.01$; ***, $p < 0.001$; NS, not significant. The box represents the median, 25th, and 75th percentiles and the error bars indicate the 5th and 95th percentiles.

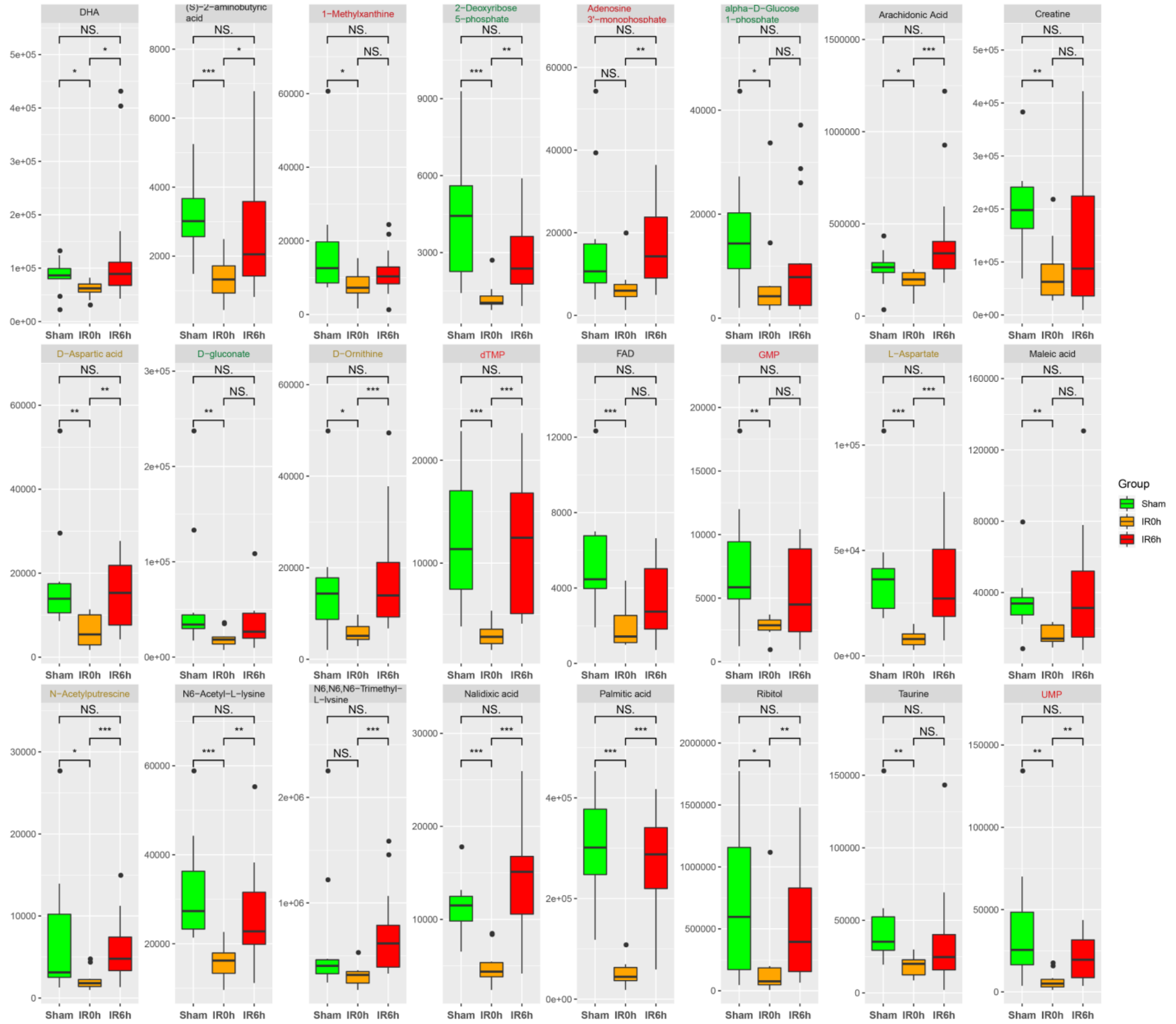

Supplementary Figure 17 Alteration of the metabolome in gut content during IIR. Metabolites which first dropped at IIR0h and then rose to normal level at IIR6h. Between group comparisons were performed using the Wilcox test; *, $p < 0.05$; **, $p < 0.01$; ***, $p < 0.001$; NS, not significant. The box represents the median, 25th, and 75th percentiles and the error bars indicate the 5th and 95th percentiles.

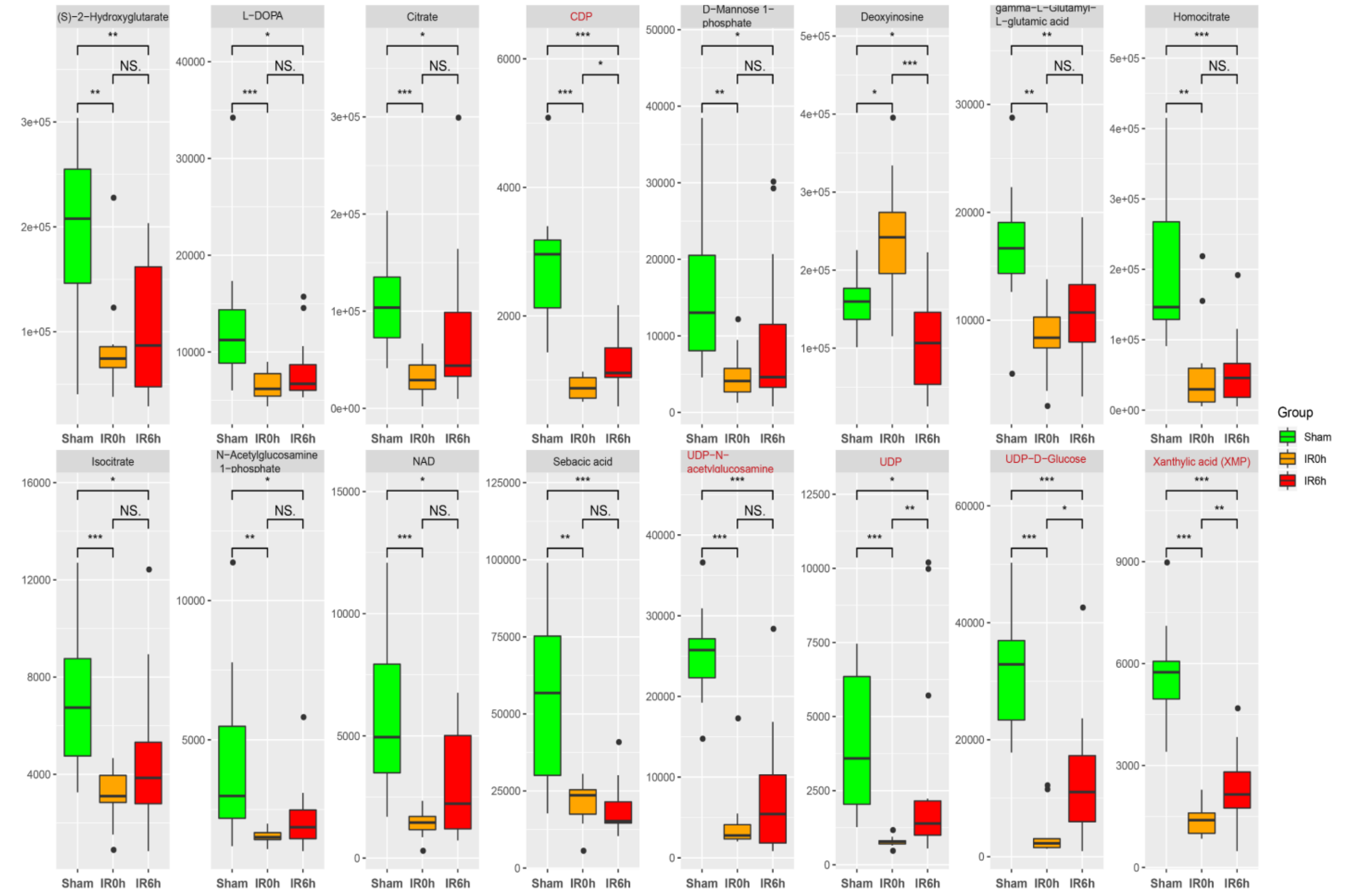

Supplementary Figure 18 Alteration of the metabolome in gut content during IIR. Metabolites which were not returned to normal levels, while significantly decreased at IIR6h compared to the Sham. Between group comparisons were performed using the Wilcox test; *, $p < 0.05$; **, $p < 0.01$; ***, $p < 0.001$; NS, not significant. The box represents the median, 25th, and 75th percentiles and the error bars indicate the 5th and 95th percentiles.

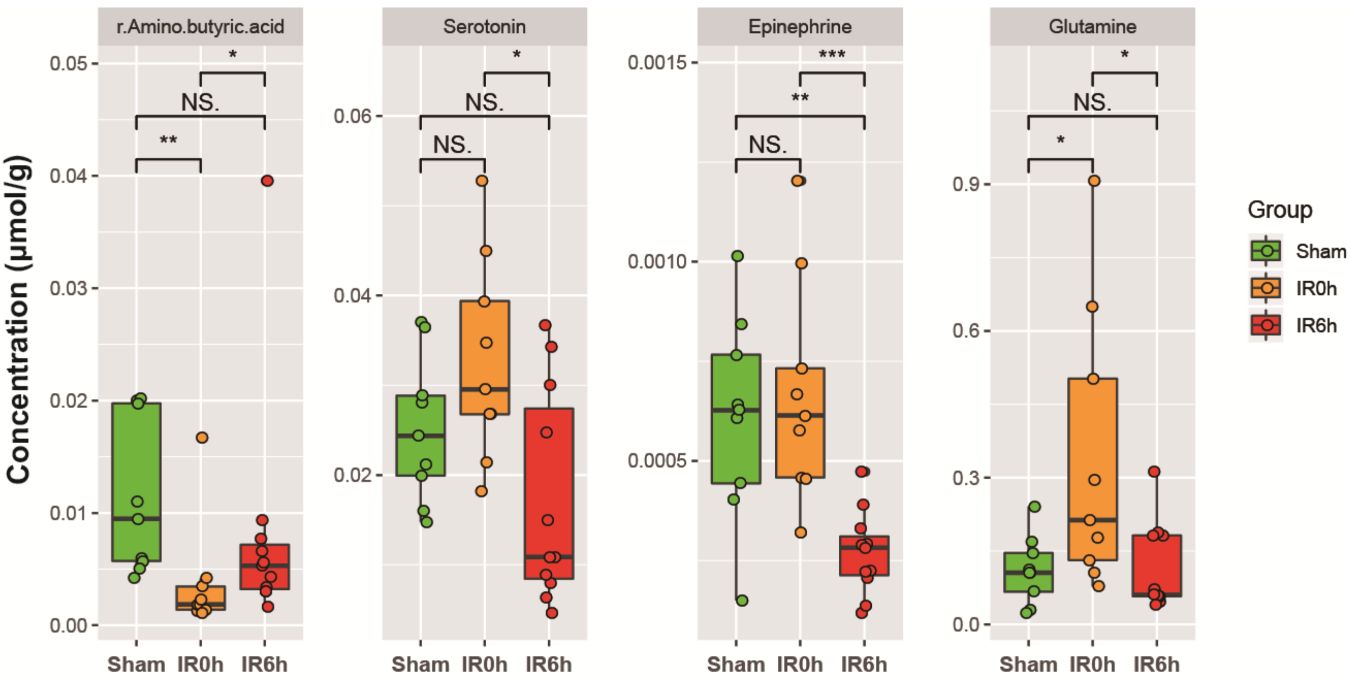

Supplementary Figure 19 Quantification of some neurotransmitters in gut content by LC/MSMS (μmol/g). Between group comparisons were performed using the Wilcox test; *, $p < 0.05$; **, $p < 0.01$; ***, $p < 0.001$; NS, not significant. The box represents the median, 25th, and 75th percentiles and the error bars indicate the 5th and 95th percentiles.

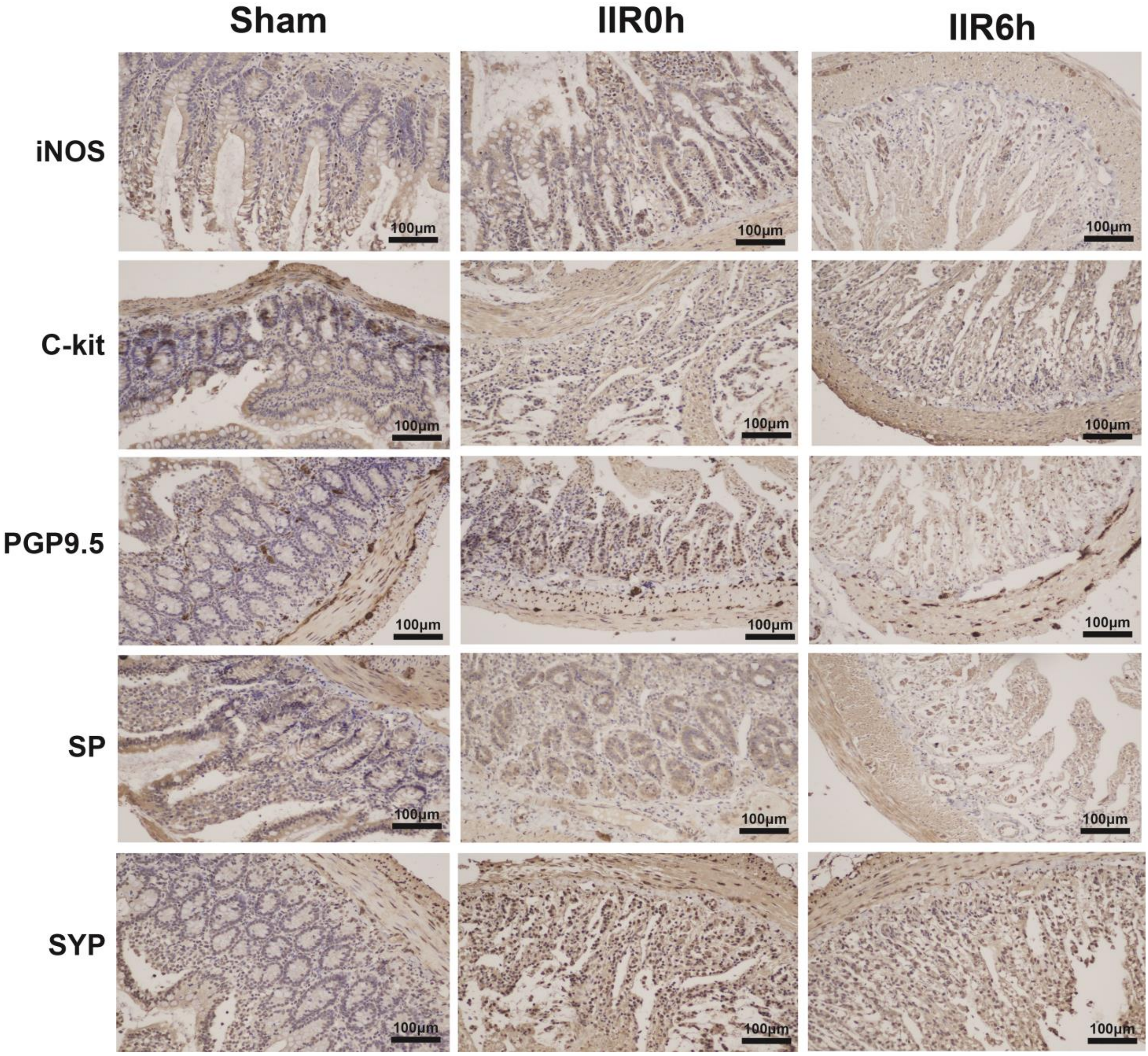

Supplementary Figure 20 Immunohistochemical level of several molecular markers for neuroendocrine system during IIR.

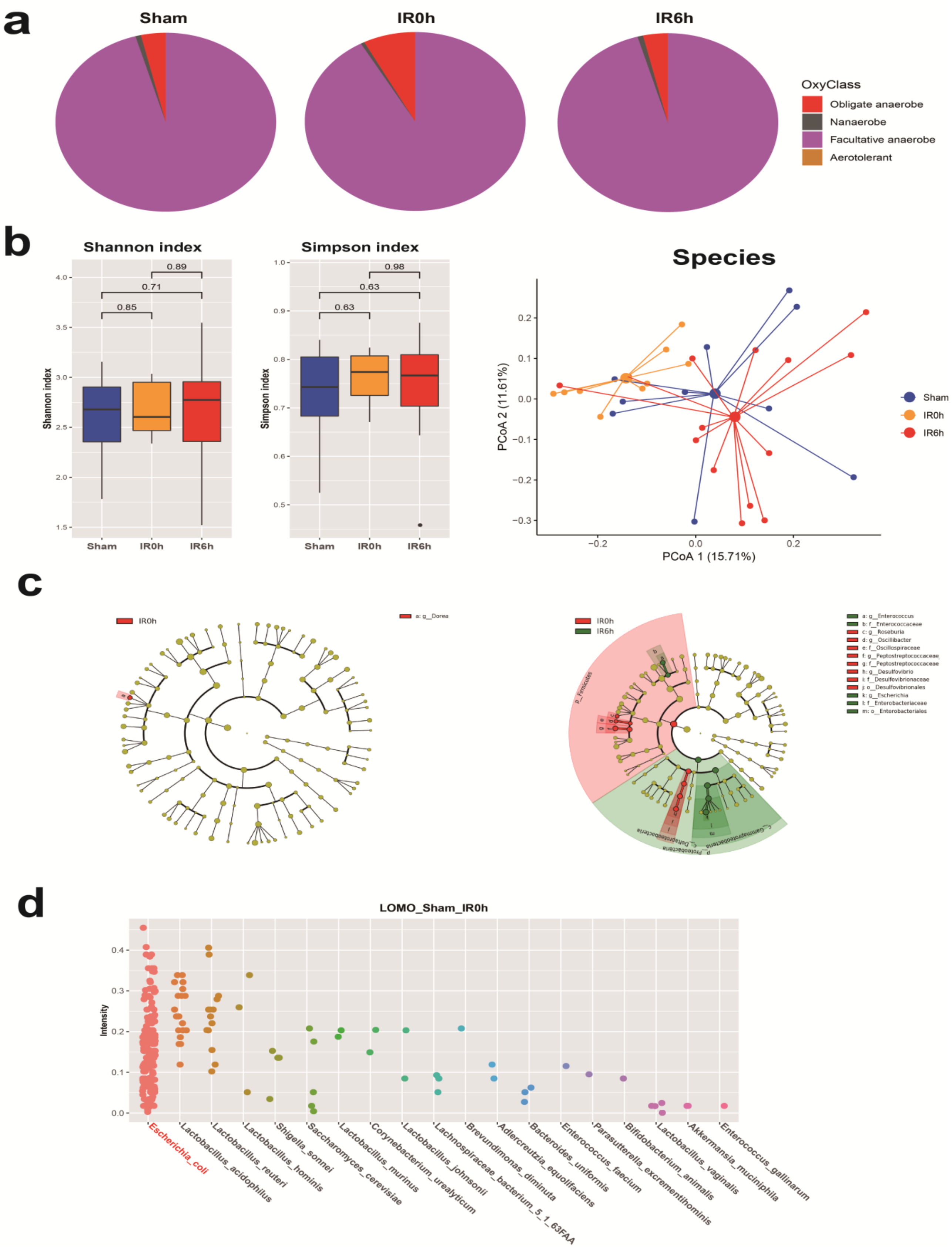

Supplementary Figure 21 Taxonomic and functional alterations of gut microbiome during IIR. **(a)** Obligate anaerobe was increased proportionally at IR0h after a period of ischemia, and then decreased in proportion at IR6h with recovered blood stream. **(b)** The microbial alpha diversity (Shannon index and Simpson index) and beta diversity among the microbial communities characterized by PCoA on weighted UniFrac distance. The box represents the median, 25th, and 75th percentiles and the error bars indicate the 5th and 95th percentiles. **(c)** Taxonomic cladogram generated from LefSe analysis showing significant difference in microbiota profile. **(d)** Escherichia coli was identified as the strongest driver specie for most of the functional pathways in ischemia period.

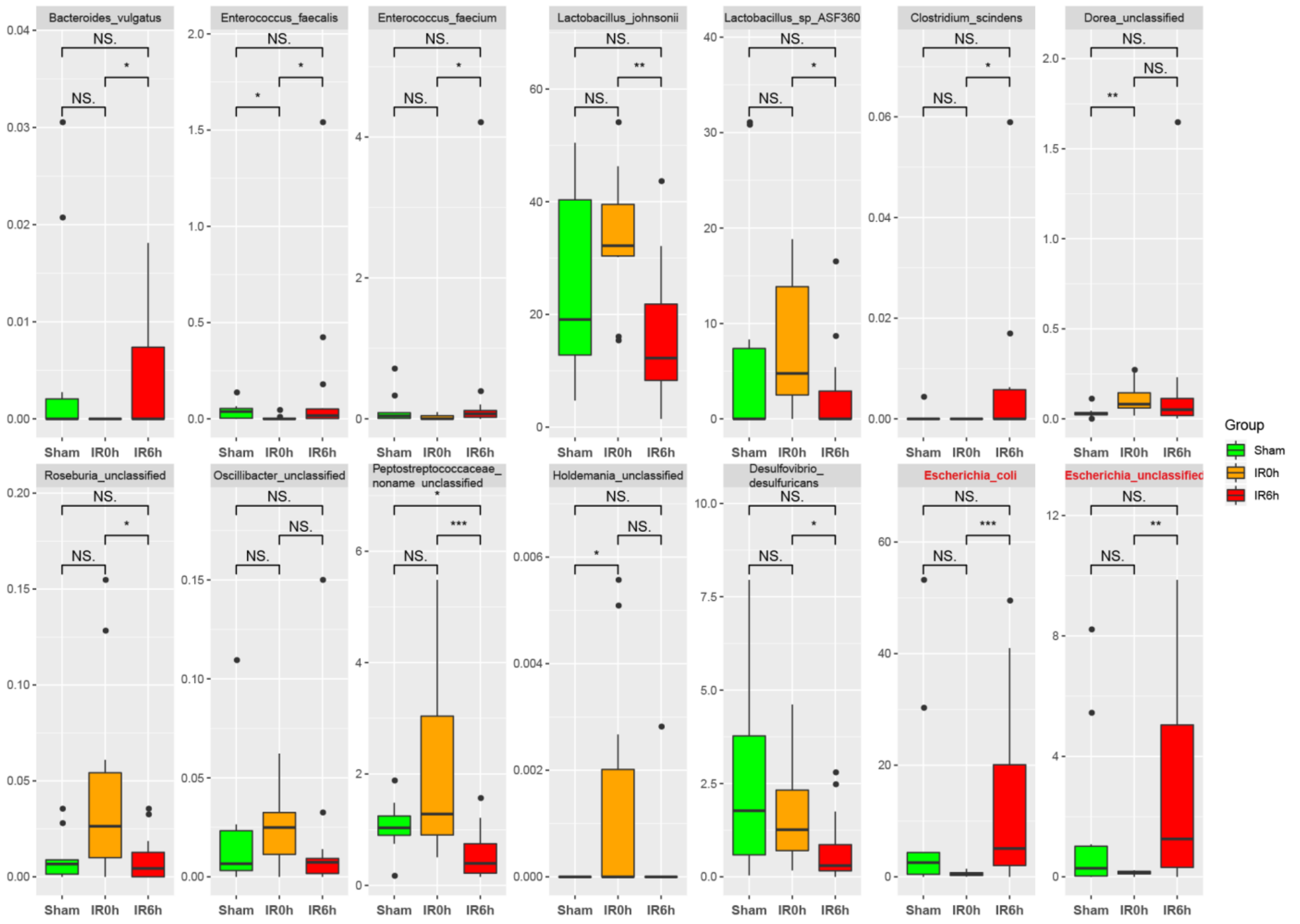

Supplementary Figure 22 Alteration of the identified different speices by LEfSe during IIR. Between group comparisons were performed using the Wilcox test; *, p < 0.05; **, p < 0.01; ***, p < 0.001; NS, not significant. The box represents the median, 25th, and 75th percentiles and the error bars indicate the 5th and 95th percentiles.

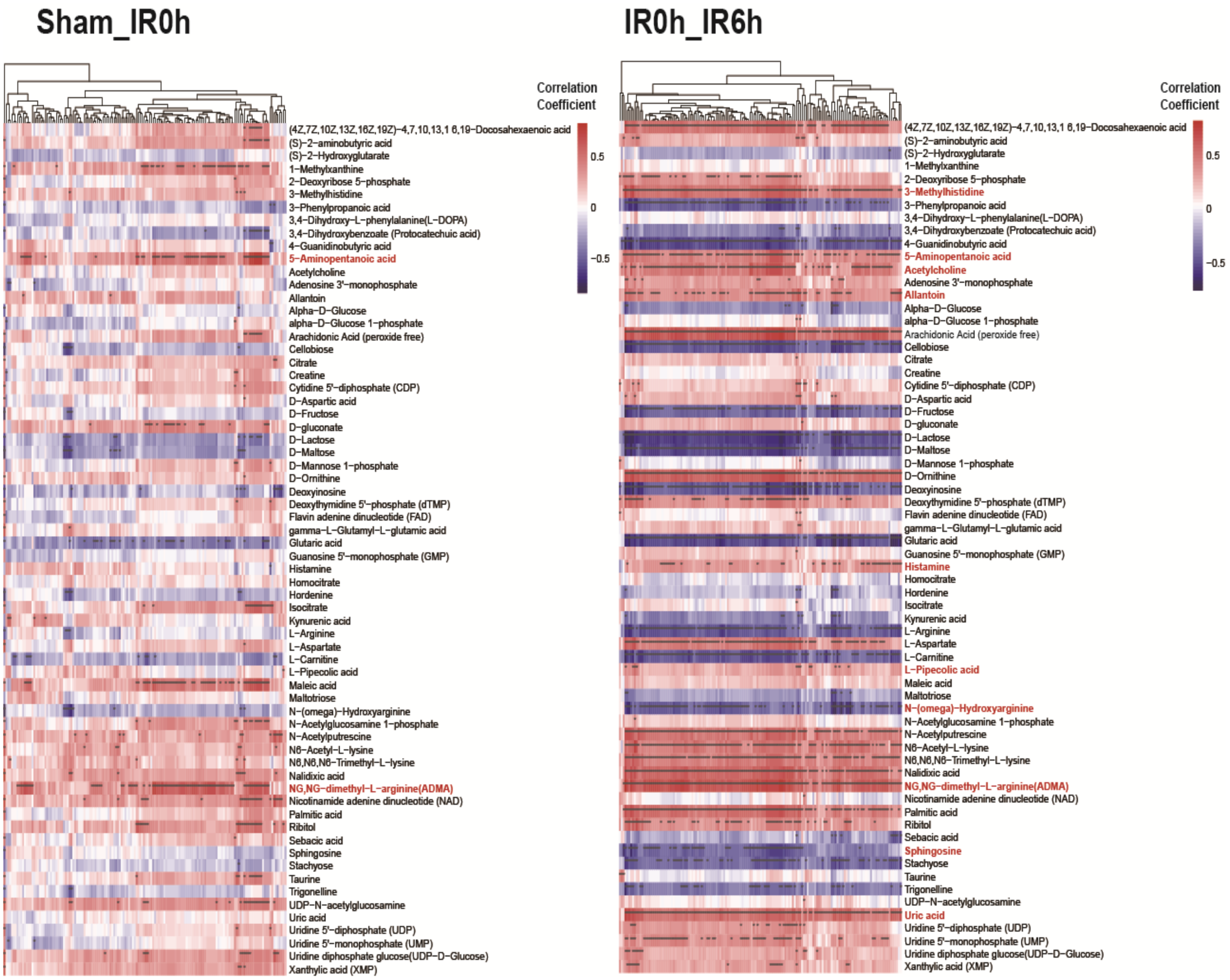

Supplementary Figure 23 Correlation links between microbial metabolites and abundances of gut pathways in reperfusion period (IR0h_IR6h) was clearly different from that in ischemia period (Sham_IR0h). Blue square indicates a reduction, white squares indicate no significant change, and red ones represent an increase. The units for the heatmap color key represent the correlation coefficient, the value of which is between -1 and 1.

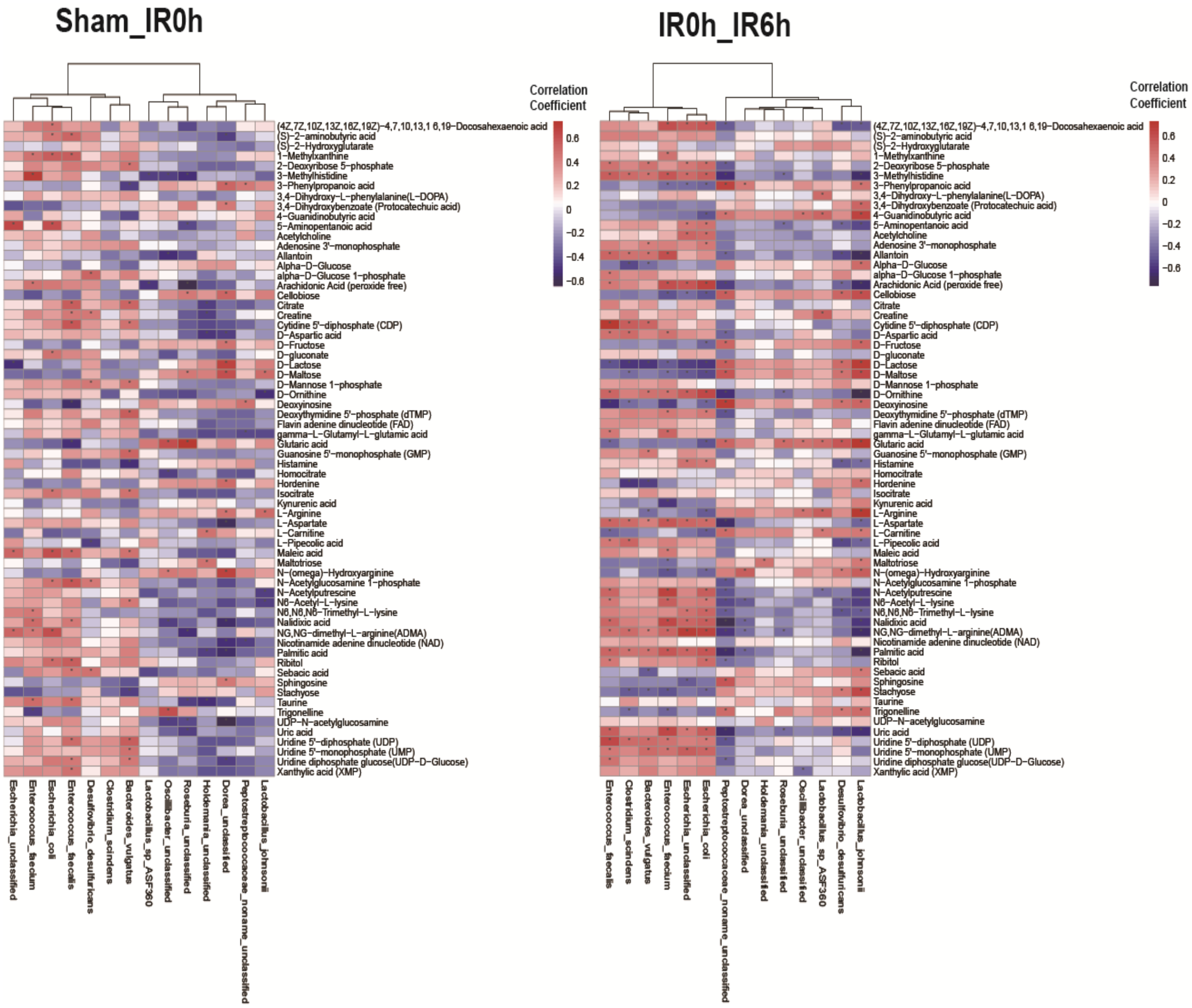

Supplementary Figure 24 Correlation links between microbial metabolites and abundances of species in reperfusion period (IR0h_IR6h) was clearly different from that in ischemia period (Sham_IR0h). Blue square indicates a reduction, white squares indicate no significant change, and red ones represent an increase. The units for the heatmap color key represent the correlation coefficient, the value of which is between -1 and 1.

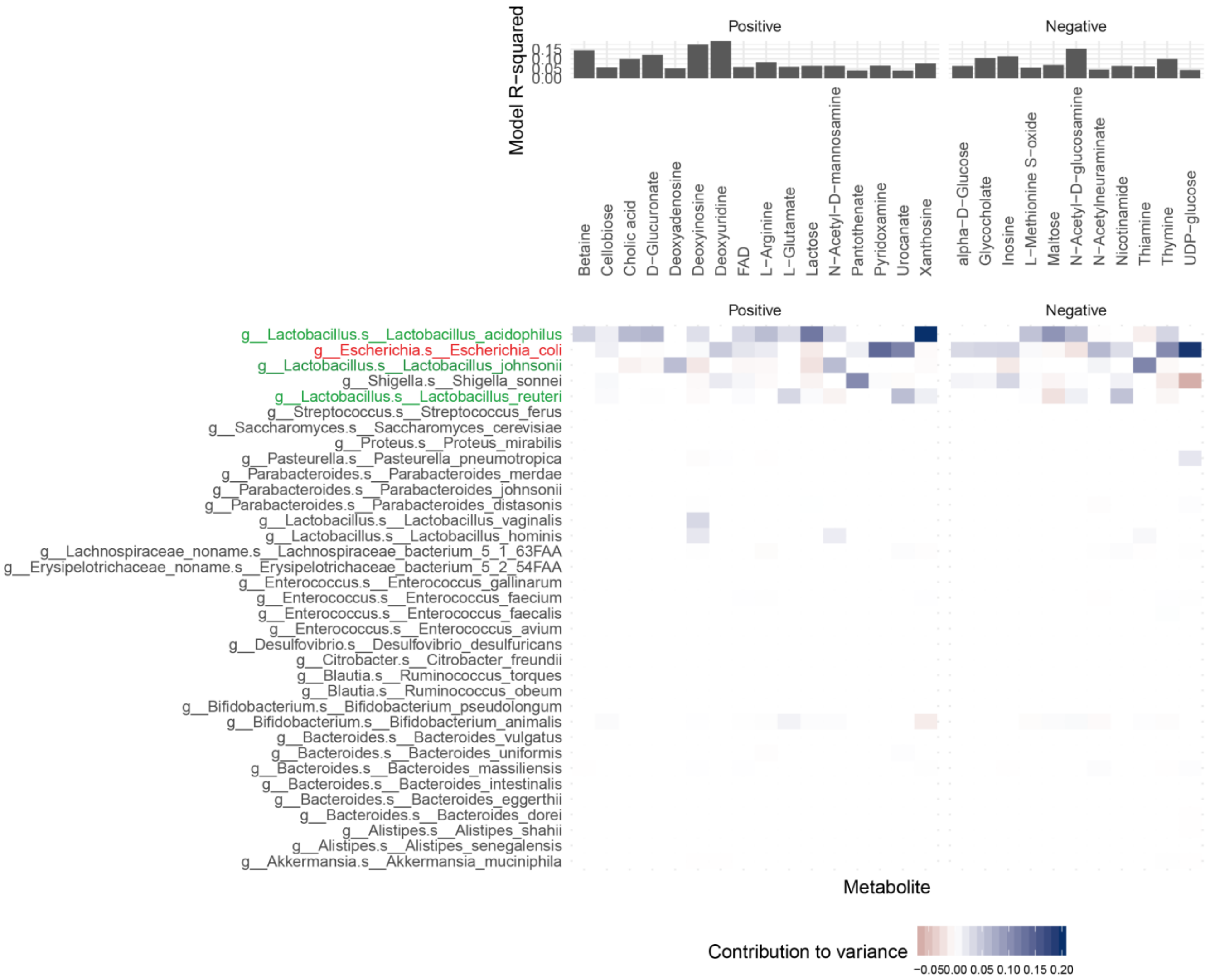

Supplementary Figure 25 Associations between metabolites and gut microbiome obtained with MIMOSA2. Blue square indicates an increasing contribution to variance, red ones represent a decreasing contribution to variance, and white squares indicate no significant change.

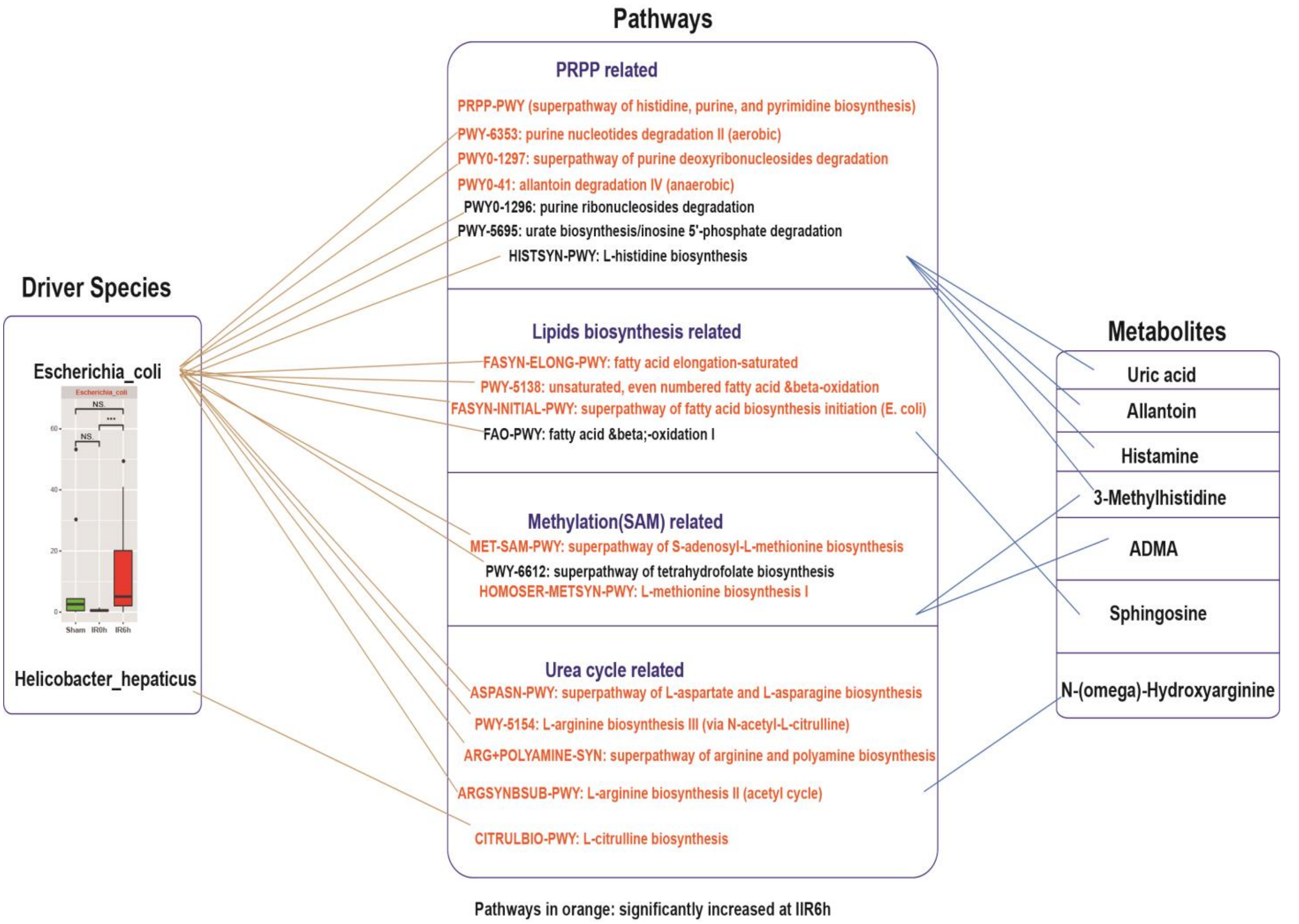

Supplementary Figure 26 Links between driver species, pathways and significantly altered metabolites in reperfusion period.
