# Supplementary material for: Integrated multi-omics reveal important roles of gut contents in intestinal ischemia–reperfusion induced injuries in rats
Source: Commun Biol. 2022 Sep 9;5:938. doi: 10.1038/s42003-022-03887-8 (PMC9463172; doi:10.1038/s42003-022-03887-8)
Supplement: Supplementary file 2 — Description of Additional Supplementary Files [file 42003_2022_3887_MOESM2_ESM.pdf]

Description of Additional Supplementary Files

File name: Supplementary Data 1
Description: Significant different metabolites identified in each tissue.

File name: Supplementary Data 2
Description: Class distribution of metabolites across tissues.

File name: Supplementary Data 3
Description: Statistics of metabolites in the gut content by a new metabolomic analysis.

File name: Supplementary Data 4
Description: Differential metabolites in gut content between Sham and IIR0h.

File name: Supplementary Data 5
Description: Differential metabolites in gut content between IIR0h and IIR6h.

File name: Supplementary Data 6
Description: Metabolites were classified into groups according to whether their abundances were significantly different between IR6h and Sham.

File name: Supplementary Data 7
Description: List of the functional bacteria.

File name: Supplementary Data 8
Description: Differential taxa identified at IR0h compared to Sham by LEfSe.

File name: Supplementary Data 9
Description: Differential taxa identified at IR6h compared to IR0h by LEfSe.

File name: Supplementary Data 10
Description: Statistics of the identified genes(KO) during IIR.

File name: Supplementary Data 11
Description: Identified differencial genes(KO) between Sham and IIR0h.

File name: Supplementary Data 12
Description: Identified differencial genes(KO) between IR0h and IR6h.

File name: Supplementary Data 13
Description: Statistics of the identified pathways during IIR.

File name: Supplementary Data 14
Description: Identified differencial pathways between Sham and IR0h.

File name: Supplementary Data 15
Description: Identified differencial pathways between IR0h and IR6h.

File name: Supplementary Data 16
Description: Microbial driver species analysis for the functional pathways using a leave-one-out algorithm between Sham and IIR0h.

File name: Supplementary Data 17
Description: Microbial driver species analysis for the functional pathways using a leave-one-out algorithm between IIR0h and IIR6h.

File name: Supplementary Data 18
Description: The MRM transitions, retention times, and conditions for quantification of neurotransmitters in gut content.

File name: Supplementary Data 19
Description: Known functions/characteristics of species obtained based on large numbers of literature retrieval.

File name: Supplementary Data 20
Description: Abundance of some bowel barrier damage related biomarkers between groups.

File name: Supplementary Data 21
Description: Pair-wise Spearman correlations as a function of time during IIR.

File name: Supplementary Data 22
Description: Gut metabolites that were significant altered during IIR and were not returned to normal levels at IIR6h.

File name: Supplementary Data 23
Description: Immunohistochemical level of several molecular markers for intestinal neuroendocrine system significantly changed during IIR.

File name: Supplementary Data 24
Description: Overall abundance changes for species with known functions and/or characteristics, including anti-inflammation, pro-inflammation and neurotransmitter producing capacity.
