# Supplementary material for: Integrated multi-omics reveal important roles of gut contents in intestinal ischemia–reperfusion induced injuries in rats
Source: Commun Biol. 2022 Sep 9;5:938. doi: 10.1038/s42003-022-03887-8 (PMC9463172; doi:10.1038/s42003-022-03887-8)
Supplement: Supplementary file 27 — Reporting Summary [file 42003_2022_3887_MOESM27_ESM.pdf]

nature portfolio

Corresponding author(s): Wei-Hua Chen, Fengguo Xu; COMMSBIO-21-0813B

Last updated by author(s): Nov 22, 2021

Reporting Summary

Nature Portfolio wishes to improve the reproducibility of the work that we publish. This form provides structure for consistency and transparency in reporting. For further information on Nature Portfolio policies, see our Editorial Policies and the Editorial Policy Checklist.

Statistics

For all statistical analyses, confirm that the following items are present in the figure legend, table legend, main text, or Methods section.

n/a	Confirmed	
☐	☒	The exact sample size (*n*) for each experimental group/condition, given as a discrete number and unit of measurement
☐	☒	A statement on whether measurements were taken from distinct samples or whether the same sample was measured repeatedly
☐	☒	The statistical test(s) used AND whether they are one- or two-sided<br>*Only common tests should be described solely by name; describe more complex techniques in the Methods section.*
☐	☒	A description of all covariates tested
☐	☒	A description of any assumptions or corrections, such as tests of normality and adjustment for multiple comparisons
☐	☒	A full description of the statistical parameters including central tendency (e.g. means) or other basic estimates (e.g. regression coefficient) AND variation (e.g. standard deviation) or associated estimates of uncertainty (e.g. confidence intervals)
☐	☒	For null hypothesis testing, the test statistic (e.g. *F*, *t*, *r*) with confidence intervals, effect sizes, degrees of freedom and *P* value noted<br>*Give P values as exact values whenever suitable.*
☒	☐	For Bayesian analysis, information on the choice of priors and Markov chain Monte Carlo settings
☐	☒	For hierarchical and complex designs, identification of the appropriate level for tests and full reporting of outcomes
☐	☒	Estimates of effect sizes (e.g. Cohen's *d*, Pearson's *r*), indicating how they were calculated

Our web collection on statistics for biologists contains articles on many of the points above.

Software and code

Policy information about availability of computer code

Data collection	no software was used
Data analysis	Bowtie2 (version bowtie2-2.1.0);MetaPhlAn2;HUMANn2;MIMOSA2;R package (version 4.0.4, http://www.r-project.org)

For manuscripts utilizing custom algorithms or software that are central to the research but not yet described in published literature, software must be made available to editors and reviewers. We strongly encourage code deposition in a community repository (e.g. GitHub). See the Nature Portfolio guidelines for submitting code & software for further information.

Data

Policy information about availability of data

All manuscripts must include a data availability statement. This statement should provide the following information, where applicable:

- Accession codes, unique identifiers, or web links for publicly available datasets
- A description of any restrictions on data availability
- For clinical datasets or third party data, please ensure that the statement adheres to our policy

The metagenomics data have been deposited into NCBI database under the BioProject accession number PRJNA669162. The authors declare that all other data supporting the findings of the study are available in the paper and online supplementary materials, or from the corresponding authors on request.

Field-specific reporting

Please select the one below that is the best fit for your research. If you are not sure, read the appropriate sections before making your selection.

☒ Life sciences ☐ Behavioural & social sciences ☐ Ecological, evolutionary & environmental sciences

For a reference copy of the document with all sections, see nature.com/documents/nr-reporting-summary-flat.pdf

Life sciences study design

All studies must disclose on these points even when the disclosure is negative.

Sample size	no sample-size calculation
Data exclusions	no data were excluded from the analyses
Replication	all attempts at replication were successful
Randomization	allocation of samples was random
Blinding	investigators were blinded to group allocation during data collection

Reporting for specific materials, systems and methods

We require information from authors about some types of materials, experimental systems and methods used in many studies. Here, indicate whether each material, system or method listed is relevant to your study. If you are not sure if a list item applies to your research, read the appropriate section before selecting a response.

Materials & experimental systems

n/a	Involved in the study	
☒	☐	Antibodies
☒	☐	Eukaryotic cell lines
☒	☐	Palaeontology and archaeology
☐	☒	Animals and other organisms
☒	☐	Human research participants
☒	☐	Clinical data
☒	☐	Dual use research of concern

Methods

n/a	Involved in the study	
☒	☐	ChIP-seq
☒	☐	Flow cytometry
☒	☐	MRI-based neuroimaging

Animals and other organisms

Policy information about studies involving animals; ARRIVE guidelines recommended for reporting animal research

Laboratory animals	Male Sprague–Dawley rats (220–260 g body weight) in specific pathogen-free (SPF) status
Wild animals	the study did not involve wild animals
Field-collected samples	Rats were randomly assigned to Sham or IIR group, maintained under controlled conditions of temperature(21 ± 1 ?) and relative humidity (&55 %) on a 12hr light/12hr dark cycle. Animals were fed ad libitum with food and water for 1 week before experiments and were fasted overnight before the day of experiment.
Ethics oversight	All animal experiments were performed in accordance with the Animal Ethics Committee guidelines of Huazhong University of science and technology.

Note that full information on the approval of the study protocol must also be provided in the manuscript.
